# Neighborhood geographic disparities in colorectal, prostate, breast, and lung cancer risk in Alabama

**DOI:** 10.1007/s11764-026-02026-0

**Published:** 2026-05-02

**Authors:** R. Blake Buchalter, Nashira I. Brown, Mahak Bhargava, Hayden D. Reeves, Syed M. Qasim Hussaini, Timiya S. Nolan, Ritu Aneja, Mackenzie E. Fowler

**Affiliations:** 1 Department of Epidemiology, School of Public Health, University of Alabama at Birmingham, Birmingham, AL USA; 2 O’Neal Comprehensive Cancer Center, Heersink School of Medicine, University of Alabama at Birmingham, Birmingham, AL USA; 3 Department of Health Behavior, School of Public Health, University of Alabama at Birmingham, Birmingham, AL USA; 4 Department of Nutrition Sciences, School of Health Professions, University of Alabama at Birmingham, Birmingham, AL USA; 5 Division of Hematology & Oncology, Department of Medicine, University of Alabama at Birmingham, Birmingham, AL USA; 6 Division of General Internal Medicine and Population Science, Department of Medicine, University of Alabama at Birmingham, Birmingham, AL USA

**Keywords:** Geographic disparities, Cancer risk, Hot spots, Area Deprivation Index, Rural cancer disparities

## Abstract

**Purpose:**

Incidence of common cancers has been rising in the U.S., with strong geographic variation in risk factors and incidence, particularly in the Southeast and in rural and high-poverty areas. Elevated cancer risk may translate into higher geographic concentrations of cancer survivors who require surveillance, follow-up care, and supportive services. Local geospatial assessment of cancer burden may inform both cancer prevention and survivorship planning.

**Methods:**

We utilized adult (≥ 18 years) incident cases of colorectal, prostate, breast, and lung cancers from the 2010–2019 Alabama Statewide Cancer Registry data. We performed Bayesian disease mapping to estimate census tract-level relative risks (RRs) and classified tracts as hot and cold spots at 99% credible intervals (CrIs). Stratified analyses examined median RR differences by 2010 Rural–Urban Commuting Area codes and 2010–2019 Area Deprivation Index (ADI) quartiles.

**Results:**

We identified 7, 9, 3, and 83 hot-spot tracts for colorectal, prostate, breast, and lung cancer incidence, respectively. Colorectal hot spots were concentrated in rural southwestern Alabama; prostate cancer hot spots formed a band across south-central Alabama; breast cancer hot spots were localized to Huntsville and Birmingham; and lung cancer hot spots were widespread across rural areas. Increasing rurality was associated with higher median RRs for colorectal, prostate, and lung cancers.

**Conclusions & Implications for Cancer Survivors:**

Neighborhood-level geographic disparities in cancer risk highlight areas with higher concentrations of cancer survivors with survivorship care needs. Linking geospatial incidence patterns with survivorship planning could support targeted strategies for surveillance, symptom management, psychosocial care, and health maintenance in high risk communities in the Deep South.

**Supplementary Information:**

The online version contains supplementary material available at https://doi.org/10.1007/s11764-026-02026-0.

## Introduction

Although cancer mortality has been decreasing in the U.S. for several decades, cancer incidence has increased for the most common cancers, including breast, prostate, and colorectal cancer (CRC) [1]. Improved screening uptake, smoking reductions, and improved treatments are key factors resulting in decreasing cancer mortality [2–4], but the causes of increasing incidence are not yet fully understood. Changes in diet, physical activity, obesity rates, environmental contaminant exposure, built environments, and other exposures over time have been postulated as potential contributors to the increasing incidence of these cancers [5–13]. Patterns in both risk factors and cancer incidence exhibit strong geographic variation, particularly in small areas such as census tracts [14, 15]. Geographic patterns of cancer risk also reflect where cancer survivors reside, creating downstream implications for local survivorship care. Neighborhood-level geospatial studies of cancer burden remain less common in the literature because of methodological complexity [16], but may help identify which local areas have higher cancer risk, which local risk factors contribute to that risk, and where demand for survivorship services is likely to be elevated [17].

The Deep South region (Alabama, Georgia, Louisiana, Mississippi, South Carolina) has profound chronic disease disparities, including disproportionately high cancer incidence rates [18–20]. The state of Alabama, in particular, has a high cancer burden, particularly for CRC, prostate, and lung cancer [21]. Excluding breast cancer (123.3 vs. 129.8 per 100,000 population), Alabama has higher age adjusted cancer incidence rates than the national average for all leading causes of cancer: prostate (116.6 vs. 113.2), CRC (40.1 vs. 36.4), and lung cancers (58.4 vs. 53.1) [21, 22]. Furthermore, Alabama has a large rural population (42%) and is highly socioeconomically deprived [23, 24]. As rural and high poverty areas in the southern U.S. have higher cancer incidence rates and cancer risk often coexists with barriers to survivorship care [25–28], Alabama is an ideal study area to identify neighborhood-level geographic patterns of cancer burden in the Deep South to provide clues about local areas with elevated needs for survivorship services.

Despite evidence of regional variations in cancer burden in Alabama [21, 23], no studies have examined how neighborhood cancer risk differs across geography, socioeconomic status, and rural/urban status. Previous studies investigating geographic differences in larger Alabama areal units (e.g., counties) [23] likely mask local heterogeneity in cancer burden. Identifying small area cancer disparities is an important step towards improved targeting of funding, clinical services, and prevention programs, as well as identifying which local exposures (e.g., point source pollution, poor food environments) may be driving the observed patterns. In this study, we used advanced geospatial analysis tools to examine neighborhood-level patterns of colorectal, prostate, breast, and lung cancer risk in Alabama. We interpret these patterns as indicators of downstream survivorship care needs, consistent with established cancer survivorship standards that emphasize coordinated follow-up care, management of persistent symptoms, psychosocial support, and health maintenance following treatment [17].

## Methods

### Study population

In this cross-sectional study, we used data from the Alabama Statewide Cancer Registry (ASCR), which is a statewide, population-based cancer registry. Among incident cases of breast (ICD-O-3-500-509), prostate (ICD-O-3-C1.9), CRC (ICD-O-3-182-199; ICD-O-3-209), and lung cancer (ICD-O-3-C34) in Alabama, we included adult (≥ 18 years) cases diagnosed between January 1, 2010, and December 31, 2019. We included only cases where the cancer was the only primary or first of multiple primary cancers diagnosed in the participant’s lifetime, including in situ cases.

### Outcome

The primary outcome was tract-level age adjusted incidence stratified by cancer type. Age adjusted expected counts by tract were calculated via direct standardization using population data stratified by 11 age categories from aggregated 2010–2019 American Community Survey data and the 2000 U.S. standard population. Tracts with zero population or zero cancer cases during the study period were excluded.

### Stratification variables

Two variables were utilized to examine stratified relationships: rural–urban status and Area Deprivation Index (ADI). Rural–urban status was measured using the 2010 Rural–Urban Commuting Area (RUCA) codes from the U.S. Department of Agriculture. RUCA codes were recategorized as metropolitan (codes 1–3), micropolitan (codes 4–6), small town (codes 7–9), and rural (code 10). We used the 2010–2019 tract-level ADI from the ‘sociome’ R package [29] and recategorized ADI into quartiles.

### Geospatial analyses

We performed geospatial disease mapping of the age adjusted incidence of CRC, prostate, breast and lung cancers across Alabama census tracts. First, we fit negative binomial hierarchical spatial Bayesian models with an offset for expected counts, based on the population size and age structure of each tract. We used a reparameterization of the Besag-York-Mollié (BYM2) approach to capture spatial dependence and local heterogeneity [30]. This form aligns with the widely used BYM family of models in small area disease‐mapping studies [31, 32], which is useful for smoothing unstable rates in small areas by borrowing strength from geographic neighbors [33]. Previous work has shown that rates based on small counts are unstable, while modeled smoothed rates can be meaningfully reported, even in small areas [34]. Posterior estimates were used to calculate relative risks (RRs) for each tract, followed by classification of tracts as hot spots (elevated risk), cold spots (reduced risk), or not significantly different based on the expected case counts at a 99% credible interval (CrI). RRs represent the extent to which the cancer risk in each tract is higher or lower than the average risk across Alabama while accounting for unmeasured spatial variation. The CrI threshold for hot and cold spots was selected to reduce false positives and provide actionable insights into the local cancer burden. We then performed stratified analyses of median RR values using recategorized 2010 RUCA codes, 2010–2019 ADI quartiles, and Holm multiple comparison adjusted non-parametric statistical tests (Kruskal–Wallis and Dunn’s test). Tract-level spatial patterns of the estimated cancer RRs, along with significant hot spots and cold spots, were visualized using thematic maps.

## Results

### Relative risk mapping

**Fig. 1:**
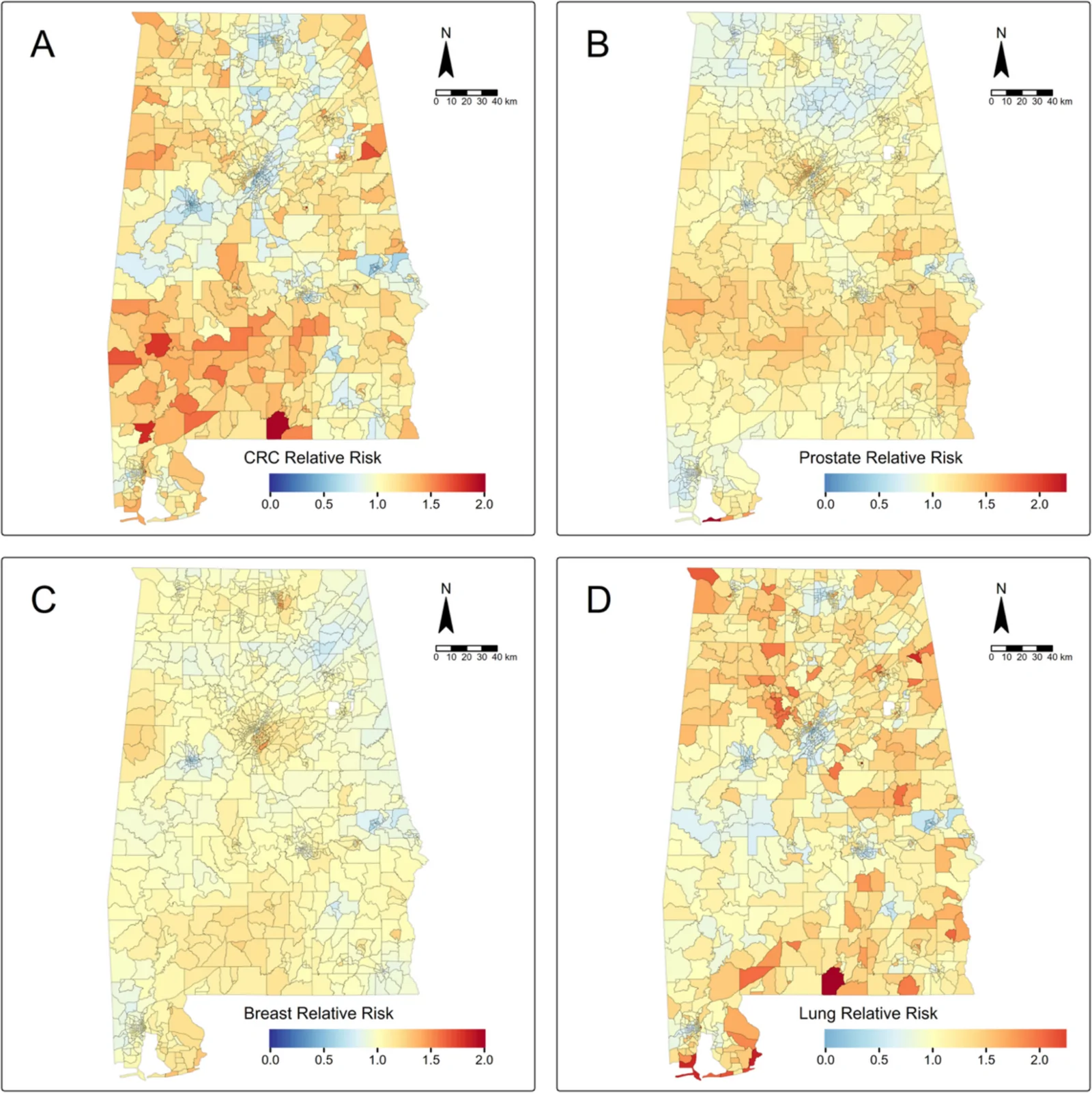
Bayesian model-derived relative risk maps for **A**) colorectal cancer, **B**) prostate cancer, **C**) breast cancer, and **D**) lung cancer incidence in Alabama census tracts

Our modeling produced baseline statewide RRs of 1.022 (95% CrI, 1.002–1.043), 1.030 (95% CrI, 1.008–1.053), 1.011 (95% CrI, 0.994–1.029), and 0.993 (95% CrI, 0.975–1.014) for colorectal, prostate, breast, and lung cancer, respectively (Tables S1–S4). Mapping of tract-specific RRs revealed elevated CRC risk (RRs = 1.5–2) in southwestern Alabama, with weaker patterns of increased risk in northwestern and east-central Alabama tracts (RRs = 1–1.5). Metropolitan tracts near Birmingham, Tuscaloosa, Huntsville, and Montgomery had reduced CRC risk, with RRs ranging from 0.5 to 1 (Fig. 1A). For prostate cancer, we observed a band of elevated risk (RRs =  ~ 1.5) across rural south-central tracts, in the northern suburban areas of Birmingham, and more elevated RRs of 1.75–2 in coastal areas near Gulf Shores (Fig. 1B). Metropolitan tracts near Mobile, Tuscaloosa, Florence, and Montgomery, and rural tracts in northern Alabama had reduced prostate cancer risk (RRs = 0.5–1). For breast cancer, there were more muted RRs than for CRC and prostate cancer. However, there was elevated risk (RRs =  ~ 1.5) in southern suburban tracts of Birmingham and eastern suburban tracts of Huntsville and a reduced risk (RRs = 0.5–0.75) in metropolitan tracts near Tuscaloosa and Auburn-Opelika (Fig. 1C). In contrast to breast cancer, RRs for lung cancer were more extreme, with elevated risk in rural north-central and southern Alabama tracts (RRs =  ~ 1.75), and particularly high risk in coastal areas near Gulf Shores (RRs =  ~ 2) (Fig. 1D). Metropolitan tracts near Birmingham, Tuscaloosa, Huntsville, and Montgomery had reduced lung cancer risk (RRs =  ~ 0.5). The full model results for each cancer can be found in Tables S1–S4.

### Hot and cold spot mapping

**Fig. 2:**
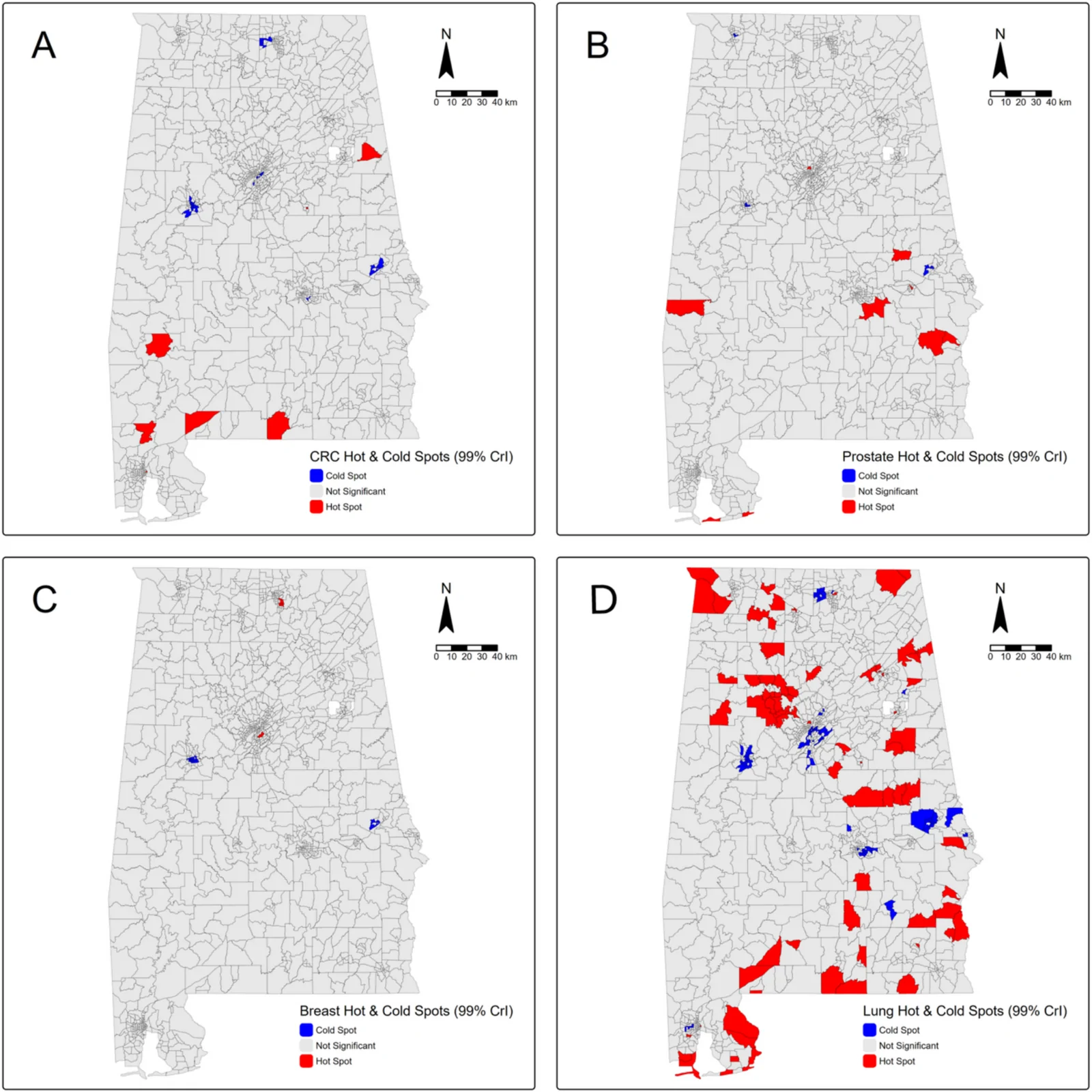
Hot-spot and cold-spot maps for **A**) colorectal cancer, **B**) prostate cancer, **C**) breast cancer, and **D**) lung cancer incidence at 99% CrI in Alabama census tracts

For CRC, prostate, breast, and lung cancer incidence, we identified 7, 9, 3, and 83 tracts as hot spots at 99% CrI, respectively. CRC hot spots were primarily in rural southwestern tracts, although two other hot-spot tracts were identified in east-central Alabama (Fig. 2A). Prostate cancer hot spots were observed across a band of tracts in south-central Alabama, with additional hot spots near coastal Gulf Shores and one tract in northern Birmingham (Fig. 2B). Breast cancer had the fewest hot spots, with two hot-spot tracts in the southern suburban areas of Huntsville and one hot-spot tract in Birmingham (Fig. 2C). In contrast, we identified the highest number of hot-spot tracts for lung cancer, which were widely distributed across Alabama but generally in more rural tracts; however, minimal hot-spot tracts were observed in northern Birmingham, southern Mobile, and northern Huntsville (Fig. 2D). We also identified cold spots for each cancer, which are shown in Fig. 2. In addition, we mapped hot spots and cold spots at 95% CrIs for a less restrictive view of the clusters (Fig. S1).

### Stratified analyses

We performed global Kruskal–Wallis tests and identified significant differences across RUCA categories and ADI quartiles for all cancers studied, indicating that pairwise Dunn tests were appropriate. Dunn pairwise comparisons of RUCA categories revealed that small town and rural tracts had significantly higher median RR values than metropolitan tracts for CRC, prostate cancer, and lung cancer. In contrast, micropolitan tracts have significantly higher median RR values for CRC and lung cancer (Table 1). In addition, there was a consistent exposure-response relationship, indicating that increasing rurality was related to increasingly elevated median RR values for CRC, prostate cancer, and lung cancer. Although we found that small town-coded tracts had significantly higher median RR values than metropolitan tracts for breast cancer, there were no significant differences between small town and rural tracts, and no evidence of an exposure-response relationship. Dunn pairwise comparisons of the ADI quartiles showed that tracts in the high deprivation quartiles (Q2, Q3, and Q4) had significantly higher RR median values than tracts in the lowest deprivation quartile for CRC and lung cancer, and the highest-deprivation quartile (Q4) had a significantly higher RR median than Q1 for prostate cancer. Notably, for breast cancer, tracts in the lowest deprivation quartile (Q1) had significantly higher median RR values than tracts in the higher-deprivation quartiles (Q2, Q3, and Q4). For ADI across all cancers studied, there was little evidence of an exposure-response relationship.

**Table 1:** Stratified relative risk medians, median differences, and Dunn’s test *p*-values for colorectal cancer, prostate cancer, breast cancer, and lung cancer incidence in Alabama census tracts

Strata	Relative risk median	Relative risk median difference	Holm-adjusted Dunn p-value
Colorectal Cancer			
Metropolitan	0.98	Ref	-
Micropolitan	1.05	0.07	** < 0.001**
Small town	1.18	0.20	** < 0.001**
Rural	1.21	0.23	** < 0.001**
ADI Q1	0.89	Ref	-
ADI Q2	1.03	0.14	** < 0.001**
ADI Q3	1.11	0.22	** < 0.001**
ADI Q4	1.09	0.20	** < 0.001**
Prostate Cancer			
Metropolitan	1.00	Ref	-
Micropolitan	1.00	0.00	0.935
Small town	1.05	0.05	**0.002**
Rural	1.10	0.10	** < 0.001**
ADI Q1	1.00	Ref	-
ADI Q2	0.96	−0.04	0.154
ADI Q3	1.00	0.00	0.778
ADI Q4	1.07	0.07	** < 0.001**
Breast Cancer			
Metropolitan	1.00	Ref	-
Micropolitan	0.96	−0.04	0.416
Small town	1.04	0.04	**0.014**
Rural	1.00	0.00	0.782
ADI Q1	1.04	Ref	-
ADI Q2	0.99	−0.05	** < 0.001**
ADI Q3	0.98	−0.06	** < 0.001**
ADI Q4	1.00	−0.04	** < 0.001**
Lung Cancer			
Metropolitan	1.00	Ref	-
Micropolitan	1.12	0.12	** < 0.001**
Small town	1.18	0.18	** < 0.001**
Rural	1.19	0.19	** < 0.001**
ADI Q1	0.80	Ref	-
ADI Q2	1.13	0.33	** < 0.001**
ADI Q3	1.20	0.40	** < 0.001**
ADI Q4	1.04	0.24	** < 0.001**

**Bold:** Holm-adjusted Dunn’s test *p* < 0.05

## Discussion

The purpose of this study was to examine census tract-level incidence rates for the four most common cancers in Alabama (CRC, prostate, lung, and breast) and to assess the influence of rurality and area deprivation on area-level incidence rates. We identified distinct neighborhood-level hot spots and cold spots of CRC, prostate, breast, and lung cancers in Alabama. From a purely geographic perspective, we found minimal overlap in hot spot patterns among the four cancers studied, suggesting that (1) differing underlying demographic factors, risk factors, or both may be influencing these patterns and (2) survivorship needs related to surveillance planning, long-term symptom burden, and supportive care are also likely to vary by cancer type and place. In contrast, there was overlap in cancer incidence cold spots, including overlapping cold spots for all four cancers in and surrounding the Tuscaloosa metropolitan area. The greatest number of hot spots was identified for lung cancer, and there was a wide geographic spread of these hot spots across rural tracts, spanning from the northern to the southern border of Alabama.

We observed concentrated CRC hot spots in rural southwestern Alabama, with additional clusters in east-central areas. Prostate cancer hot spots were observed in south-central Alabama, with additional clusters near coastal Gulf Shores and a tract in northern Birmingham. Breast cancer had the fewest hot spots, reflecting fewer extreme RRs, with two tracts in the southern suburban areas of Huntsville and one in Birmingham. Conversely, lung cancer exhibited the greatest number of hot spots, spread widely across Alabama. These tracts were predominantly in rural areas, with smaller clusters observed in northern Birmingham, southern Mobile, and northern Huntsville. Identifying these local hot spots provides valuable information about factors that potentially influence differences in cancer risk, such as smoking status, obesity, physical inactivity, screening rates, and environmental factors that may be driving cancer-specific trends [35, 36].

To better understand the potential drivers behind the observed hot spot patterns, we considered existing data on geographic patterns in cancer risk factors. Data from the Cancer InFocus platform in Alabama, for instance, demonstrate geographic patterns across census tracts for smoking status, obesity, and physical inactivity (Figs. S4–S6) [37]. Also available is the distribution of breast cancer screening rates and CRC screening rates across census tracts and Alabama (Figs. S7–S8) and the distribution of natural and built environmental factors such as the proportion of individuals lacking reliable transportation or experiencing food insecurity (Figs. S10–S11), the concentration of particulate matter 2.5 (PM2.5), and locations of toxic release inventory facilities (Figs. S12–S13). In general, the geographic distribution of adverse levels of these factors closely follows the RRs for the incidence of all cancer types examined. However, no correlation analyses were performed. Moreover, there is overlap between the hot spots identified for breast cancer and areas with higher smoking rates, obesity, lack of physical activity, or food insecurity, which can contribute to breast cancer risk [38, 39]. Similar patterns were seen for prostate cancer and CRC hot spots [40, 41]. For lung cancer, there was some overlap between areas with higher smoking rates. Interestingly, only some areas with higher PM2.5 concentrations were lung cancer hot spots, but there was partial overlap with locations of toxic release inventory facilities [42–44]. Further information about the toxic substances released from these locations, the concentrations released from these facilities, and how long they have been in the community may elucidate some of the potential reasons for the observed lung cancer hot spots. Importantly, these contextual risks persist beyond diagnosis, shaping survivorship experiences by influencing long-term symptom burden, risk of recurrence or second primary cancers, and the need for ongoing health maintenance and supportive care in high-risk neighborhoods [45–47].

Our findings indicate notable disparities in median RR values across cancer types in relation to rurality. For CRC, prostate, and lung cancer, increasing rurality was associated with higher median RR values, suggesting a possible exposure-response relationship and aligning with prior research showing higher rates of CRC and lung cancer in rural populations compared with metropolitan areas [25]. This trend did not hold for breast cancer, where evidence suggests that incidence is lower in rural areas (104.4 per 100,000) than in metropolitan areas (110.8 per 100,000), based on the most recent trends [25]. Our analysis also revealed that, compared with metropolitan areas, micropolitan tracts exhibited higher median RR values for CRC and lung cancer, and small town tracts showed higher median RR values for breast cancer. However, these differences were not statistically significant. Consistent with previous findings [27], breast cancer incidence was lower in rural tracts than in metropolitan tracts. We postulate that these lower rates may be associated with women in rural areas being less likely to undergo mammography [48]. Observed patterns for CRC and lung cancer may be driven by differential screening uptake, environmental exposures (e.g., air pollution), and higher smoking prevalence in rural areas [49–53]. These rural structural conditions may also affect survivorship care delivery, including coordination of clinical surveillance, management of long-term symptoms, and access to psychosocial and supportive services following treatment [28]. Combined with disparities in cancer risk factor education, lifestyle behaviors comprising poor diet, reduced physical activity, and inadequate weight management may further compound inequities in cancer incidence and survivorship [54, 55].

Consistent with previous studies [56], we observed disparities in cancer incidence associated with Area Deprivation Index. Particularly, higher deprivation was linked to increased incidence of CRC and lung cancers, along with elevated RR for prostate cancer. Breast cancer showed the opposite trend, with individuals in the lowest deprivation quartile experiencing significantly higher RR compared with those in more deprived areas. However, these results are consistent with those of prior studies, indicating that triple negative breast cancer is less affected by social factors than hormone-positive breast cancer [57]. We were unable to calculate incidence by breast cancer subtype in this study, but these results are likely driven by a single subtype [58, 59]. Examining data from Cancer InFocus, there is geographic overlap between census tracts with higher Social Vulnerability Index (a measure similar to ADI) and the census tracts with higher cancer risk in Alabama (Fig. S9) [37].

Our findings have several implications for cancer prevention, control, and survivorship in Alabama. Targeted education and outreach efforts in hot spot areas, implemented through partnerships with sectors such as the Alabama Department of Public Health, the University of Alabama at Birmingham O’Neal Comprehensive Cancer Center Office of Community Outreach and Engagement, and community-based organizations, could address disparities across the cancer control continuum. For example, mailed fecal screening programs could be piloted in the seven tracts identified as CRC incidence hot spots for maximum impact [60]. In these neighborhoods, community health worker/navigator programs, satellite survivorship clinics, telehealth, and transportation improvements could be targeted to support CRC survivors [61–64]. Other potential interventions include mobile screening units for prostate and lung cancers [65]; patient navigation for those with positive screening tests or ongoing care needs [66]; education on cancer risk factors and the importance of follow-up after diagnosis and treatment [67]; and transportation infrastructure improvements to increase access to primary care, supportive services, and opportunities to engage in healthy behaviors such as physical activity and purchasing nutritious foods [68].

### Limitations

Identifying cancer incidence hot spots using our approach has several limitations. We simplified our model results into a binary framework, which can conceal uncertainty about area-specific risks. Our choice of CrIs could be viewed as arbitrary, and small changes to this level can lead to different areas being labeled as significant. The 99% CrI threshold was selected based on our primary goal of identifying local areas with the highest risk to inform cost-effective targeting of preventive pilot programs. We also produced results for 95% CrIs and generally found similar hot and cold spot patterns in comparison to 99% CrIs (Fig. S1). Because the BYM2 model introduces spatial correlation, nearby areas may appear to have a high risk due to smoothing rather than reflecting a truly elevated local risk. The model’s shrinkage toward the mean can also obscure real hot spots in areas with small populations or limited data. For these reasons, results based on CrIs should be interpreted carefully. In addition, we were unable to perform race-stratified analyses because of insufficient geographic contiguity after stratification. Racial subgroups are distributed unequally across the state, contributing to this lack of contiguity (Figs. S2–S3). Because BYM2 models rely on the structure of the spatial adjacency graph, the spatial random effect (i.e., how we computed RRs) is most interpretable when the graph is well-connected. Extensive missingness in areal units can fragment the graph into disconnected components, altering neighborhood relationships and requiring special handling for valid model interpretation [69]. As certain demographic subsets had zero cancer cases across many concurrent tracts (e.g., zero CRC cases among the non-Hispanic Black population in many northern Alabama tracts), it was not feasible to perform spatial modeling stratified by race. Another limitation is that, due to the ecological nature of the study, our results may not reflect individual-level risk. However, our findings make novel contributions to the literature by estimating the small area risk for statewide cancer datasets.

Future research should prioritize understanding how place shapes cancer survivorship experiences and outcomes in high cancer risk neighborhoods. Linking risk patterns to survivorship outcomes such as continuity of follow-up care, management of long-term symptoms, quality of life, and risks of recurrence and second primary cancers would provide critical insight into how geographic context influences survivorship trajectories. Qualitative and mixed-methods studies are needed to identify contextual barriers to survivorship care, including transportation, access to psychosocial and supportive services, and coordination of care following treatment, particularly in rural and disadvantaged areas. Combining geospatial findings with survivorship data will help inform place-based survivorship planning and resource allocation and support more equitable delivery of survivorship care across the Deep South.

### Conclusions & implications for cancer survivors

There are geographic disparities in Alabama for cancers with the highest incidence in the U.S., particularly in more rural and socioeconomically deprived neighborhoods. These disparities likely extend beyond diagnosis and also exist for cancer outcomes and survivorship [46, 70]. Rural and deprived areas face barriers to timely follow-up care, rehabilitation, and access to supportive services, exacerbating disparities in treatment adherence, symptom management, and patient quality of life [28, 71–73]. Integrating geospatial methods into survivorship planning could enable improved intervention targeting, such as mobile survivorship clinics, telehealth-based counseling, and community health worker navigation programs in high risk neighborhoods [74–76]. Survivorship resources could be linked with local public health infrastructure and cancer center outreach efforts in Alabama and other areas in the Deep South to help reduce local gaps in surveillance for recurrence, address psychosocial needs, and improve long-term outcomes for cancer survivors in underserved neighborhoods.

## Supplementary Information

Below is the link to the electronic supplementary material.

ESM 1 (PDF 682 KB)

## Data Availability

Data supporting these findings were used under license and are available upon request to the Alabama State Cancer Registry at the Alabama Department of Public Health. For supplementary figures using Cancer InFocus data, Cancer InFocus is data-gathering and visualization software developed by the University of Kentucky Markey Cancer Center and was made available to the UAB O’Neal Comprehensive Cancer Center through a no-cost licensing agreement.

## References

[1] Siegel RL, Kratzer TB, Giaquinto AN, Sung H, Jemal A. Cancer statistics, 2025. CA Cancer J Clin. 2025;75:10–45. 10.3322/caac.21871. PMC11745215 39817679

[2] Goddard KAB, Feuer EJ, Mandelblatt JS, Meza R, Holford TR, Jeon J, et al. Estimation of cancer deaths averted from prevention, screening, and treatment efforts, 1975–2020. JAMA Oncol. 2025;11:162–7. 10.1001/jamaoncol.2024.5381. PMC11622066 39636625

[3] Jemal A, Ward E, Thun M. Declining death rates reflect progress against cancer. PLoS One. 2010;5:e9584. 10.1371/journal.pone.0009584. PMC2834749 20231893

[4] Shiels MS, Freedman ND, Haque AT, Berrington de González A, Lipkowitz S, Lowy DR, et al. US cancer deaths prevented due to survival improvements stratified by extent of disease, 2010-2019. J Natl Cancer Inst. 2025;117:2089–95. 10.1093/jnci/djaf192. PMC12505133 40668759

[5] Sezavar AH, Rastegar-Pouyani N, Rahimi Kakavandi N, Fakhari F, Jafarzadeh E, Aliebrahimi S, et al. Examining the relationship between per-and polyfluoroalkyl substances and breast, colorectal, prostate, and ovarian cancers: a meta-analysis. Crit Rev Toxicol. 2024;54:981–95. 10.1080/10408444.2024.2425669. 39636584

[6] Esposito K, Chiodini P, Colao A, Lenzi A, Giugliano D. Metabolic syndrome and risk of cancer: a systematic review and meta-analysis. Diabetes Care. 2012;35:2402–11. 10.2337/dc12-0336. PMC3476894 23093685

[7] Motterle G, DE Zorzi L, Zecchini G, Mandato FG, Ferraioli G, Bianco M, et al. Metabolic syndrome and risk of prostate cancer: a systematic review and meta-analysis. Panminerva Med. 2022;64:337–43. 10.23736/S0031-0808.21.04507-9. 34859640

[8] DeRouen MC, Tao L, Shariff-Marco S, Yang J, Shvetsov YB, Park S-Y, et al. Neighborhood obesogenic environment and risk of prostate cancer: the multiethnic cohort. Cancer Epidemiol Biomarkers Prev. 2022;31:972–81. 10.1158/1055-9965.EPI-21-1185. PMC9074096 35506246

[9] Conroy SM, Clarke CA, Yang J, Shariff-Marco S, Shvetsov YB, Park S-Y, et al. Contextual impact of neighborhood obesogenic factors on postmenopausal breast cancer: the multiethnic cohort. Cancer Epidemiol Biomarkers Prev. 2017;26:480–9. 10.1158/1055-9965.EPI-16-0941. PMC5380519 28143808

[10] Ungvari Z, Fekete M, Varga P, Lehoczki A, Munkácsy G, Fekete JT, et al. Association between red and processed meat consumption and colorectal cancer risk: a comprehensive meta-analysis of prospective studies. Geroscience. 2025;47:5123–40. 10.1007/s11357-025-01646-1. PMC12181564 40210826

[11] Wu Y, Zhang D, Kang S. Physical activity and risk of breast cancer: a meta-analysis of prospective studies. Breast Cancer Res Treat. 2013;137:869–82. 10.1007/s10549-012-2396-7. 23274845

[12] Shaw E, Farris MS, Stone CR, Derksen JWG, Johnson R, Hilsden RJ, et al. Effects of physical activity on colorectal cancer risk among family history and body mass index subgroups: a systematic review and meta-analysis. BMC Cancer. 2018;18:71. 10.1186/s12885-017-3970-5. PMC5763991 29325535

[13] Nouri-Majd S, Salari-Moghaddam A, Aminianfar A, Larijani B, Esmaillzadeh A. Association between red and processed meat consumption and risk of prostate cancer: a systematic review and meta-analysis. Front Nutr. 2022;9:801722. 10.3389/fnut.2022.801722. PMC8859108 35198587

[14] Bobbitt JR, Liu F, Keri RA, Cullen J. Cancer burden in neighborhoods with greater racial diversity and environmental burden. JAMA Netw Open. 2025;8:e2516740. 10.1001/jamanetworkopen.2025.16740. PMC12551422 40540270

[15] Torres AZ, Phelan-Emrick D, Castillo-Salgado C. Evaluating neighborhood correlates and geospatial distribution of breast, cervical, and colorectal cancer incidence. Front Oncol. 2018. 10.3389/fonc.2018.00471. PMC6218580 30425965

[16] Centers for Disease Control and Prevention. Investigating cancer clusters and unusual patterns of cancer: Challenges and limitations [Internet]. Department of Health and Human Services; 2022. https://www.cdc.gov/cancer-environment/media/pdfs/Challenges-and-Limitations-508.pdf?

[17] Mollica MA, McWhirter G, Tonorezos E, Fenderson J, Freyer DR, Jefford M, et al. Developing national cancer survivorship standards to inform quality of care in the United States using a consensus approach. J Cancer Surviv. 2024;18:1190–9. 10.1007/s11764-024-01602-6. PMC11324674 38739299

[18] Parcha V, Kalra R, Suri SS, Malla G, Wang TJ, Arora G, et al. Geographic variation in cardiovascular health among American adults. Mayo Clin Proc. 2021;96:1770–81. 10.1016/j.mayocp.2020.12.034. PMC8260439 33775420

[19] Islami F, Miller KD, Jemal A. Cancer burden in the United States—a review. Annals of Cancer Epidemiology. 2018. 10.21037/ace.2018.08.02.

[20] DuBois TD. Geographic disparities in cancer incidence in the US population aged 20 to 49 years, 2016–2020. Prev Chronic Dis. 2024. 10.5888/pcd21.230335. PMC11086694 38723272

[21] NCI. State Cancer Profiles, Incidence Rate Tables 2017–2021 [Internet]. 2025. https://statecancerprofiles.cancer.gov/data-topics/incidence.html

[22] Alabama Statewide Cancer Registry. Alabama Cancer Statistics: A sourcebook of cancer data for cancer prevention and control activities in Alabama (2011–2020) [Internet]. 2023. https://www.alabamapublichealth.gov/tobacco/assets/acs2023.pdf

[23] Alabama Department of Public Health. Alabama Cancer Statistics 2023: A Sourcebook of Cancer Data for Cancer Prevention and Control Activities in Alabama [Internet]. 2023. https://www.alabamapublichealth.gov/tobacco/assets/acs2023.pdf

[24] United For ALICE, United Ways of Alabama. ALICE in Alabama: A Study of Financial Hardship [Internet]. 2025. https://www.unitedforalice.org/Attachments/AllReports/state-of-alice-report-alabama-2025.pdf

[25] Semprini J, Gadag K, Williams G, Muldrow A, Zahnd WE. Rural–urban cancer incidence and trends in the United States, 2000 to 2019. Cancer epidemiology, biomarkers & prevention. Am Assoc Cancer Res. 2024;33:1012–22. 10.1158/1055-9965.EPI-24-0072 38801414

[26] Boscoe FP, Henry KA, Sherman RL, Johnson CJ. The relationship between cancer incidence, stage and poverty in the United States. Int J Cancer. 2016;139:607–12. 10.1002/ijc.30087. 26991033

[27] Zahnd WE, James AS, Jenkins WD, Izadi SR, Fogleman AJ, Steward DE, et al. Rural-urban differences in cancer incidence and trends in the United States. Cancer Epidemiology and Prevention Biomarkers. 2017;cebp. 0430.2017. 10.1158/1055-9965.EPI-17-0430 PMC5787045 28751476

[28] Tarver WL, Justice Z, Jonnalagadda P, Rahurkar S, Obeng-Gyasi S, Krok-Schoen JL, et al. A scoping review of the evidence on survivorship care plans among minority, rural, and low-income populations. J Cancer Surviv. 2025;19:1956–94. 10.1007/s11764-024-01609-z. PMC12546521 38907799

[29] Berg KA, Dalton JE, Gunzler DD, Coulton CJ, Freedman DA, Krieger NI, et al. The ADI-3: a revised neighborhood risk index of the social determinants of health over time and place. Health Services and Outcomes Research Methodology. Springer; 2021;1–24. 10.1007/s10742-021-00248-6.

[30] Riebler A, Sørbye SH, Simpson D, Rue H. An intuitive Bayesian spatial model for disease mapping that accounts for scaling. Statistical methods in medical research. Sage Publ Sage UK: London, England. 2016;25:1145–65. 10.1177/0962280216660421 27566770

[31] Duncan EW, White NM, Mengersen K. Spatial smoothing in Bayesian models: a comparison of weights matrix specifications and their impact on inference. Int J Health Geogr. 2017;16:47. 10.1186/s12942-017-0120-x. PMC5732501 29246157

[32] Goungounga JA, Gaudart J, Colonna M, Giorgi R. Impact of socioeconomic inequalities on geographic disparities in cancer incidence: comparison of methods for spatial disease mapping. BMC Medical Research Methodology. Springer; 2016;16:136. 10.1186/s12874-016-0228-x PMC5059978 27729017

[33] Besag J, York J, Mollié A. Bayesian image restoration, with two applications in spatial statistics. Annals of the institute of statistical mathematics. Springer; 1991;43:1–20.

[34] Gause EL, Schumacher AE, Ellyson AM, Withers SD, Mayer JD, Rowhani-Rahbar A. An introduction to Bayesian spatial smoothing methods for disease mapping: modeling county firearm suicide mortality rates. Am J Epidemiol. 2024;kwae005. 10.1093/aje/kwae005. PMC12096304 38375682

[35] Guo LR, Hughes MC, Wright ME, Harris AH, Osias MC. Geospatial hot spots and cold spots in US cancer disparities and associated risk factors, 2004-2008 to 2014-2018. Prev Chronic Dis. 2024;21:E84. 10.5888/pcd21.240046. PMC11567514 39481013

[36] Luo C, Khan S, Jin L, James AS, Colditz GA, Drake BF. Where should the cancer control interventions target: a geospatial hotspot analysis for major cancer mortality 2018 to 2022 in the United States. Cancer Epidemiol Biomarkers Prev. 2025;34:1074–9. 10.1158/1055-9965.EPI-24-0957. PMC12137688 39636168

[37] Burus T, McAfee CR, Wilhite NP, Hull PC. Measuring the impact of a catchment area surveillance tool on cancer center adopters. Cancer. 2025;131:e35710. 10.1002/cncr.35710. PMC11725053 39799407

[38] Cohen SY, Stoll CR, Anandarajah A, Doering M, Colditz GA. Modifiable risk factors in women at high risk of breast cancer: a systematic review. Breast Cancer Res. 2023;25:45. 10.1186/s13058-023-01636-1. PMC10123992 37095519

[39] Coughlin SS. Social determinants of breast cancer risk, stage, and survival. Breast Cancer Res Treat. 2019;177:537–48. 10.1007/s10549-019-05340-7. 31270761

[40] Ziglioli F, Patera A, Isgrò G, Campobasso D, Guarino G, Maestroni U. Impact of modifiable lifestyle risk factors for prostate cancer prevention: a review of the literature. Front Oncol. 2023;13:1203791. 10.3389/fonc.2023.1203791. PMC10515617 37746271

[41] Roshandel G, Ghasemi-Kebria F, Malekzadeh R. Colorectal cancer: epidemiology, risk factors, and prevention. Cancers. 2024;16:1530. 10.3390/cancers16081530. PMC11049480 38672612

[42] Alberg AJ, Samet JM. Epidemiology of lung cancer. Chest. 2003;123:21S-49S. 10.1378/chest.123.1_suppl.21s. 12527563

[43] Huang F, Pan B, Wu J, Chen E, Chen L. Relationship between exposure to PM2.5 and lung cancer incidence and mortality: a meta-analysis. Oncotarget. 2017;8:43322–31. 10.18632/oncotarget.17313. PMC5522148 28487493

[44] Luo J, Hendryx M, Ducatman A. Association between six environmental chemicals and lung cancer incidence in the United States. J Environ Public Health. 2011;2011:463701. 10.1155/2011/463701. PMC3136160 21776439

[45] Balogun Z, Gardiner LA, Li J, Moroni EA, Rosenzweig M, Nilsen ML. Neighborhood deprivation and symptoms, psychological distress, and quality of life among head and neck cancer survivors. JAMA Otolaryngol-Head Neck Surg. 2024;150:295–302. 10.1001/jamaoto.2023.4672. PMC10884950 38386337

[46] Abdullah A, Liu Z, Molinari M. From diagnosis to survivorship: the role of social determinants in cancer care. Cancers. 2025;17:1067. 10.3390/cancers17071067. PMC11987860 40227566

[47] Lo S, Li Y, Laden F, Kessler W, Lanuti M, Gainor JF, et al. Neighborhood socioeconomics and lung cancer recurrence and progression. Clinical Lung Cancer [Internet]. Elsevier; [cited 2026 Apr 1]; 10.1016/j.cllc.2026.03.011 42036275

[48] Tran L, Tran P. US urban–rural disparities in breast cancer-screening practices at the national, regional, and state level, 2012–2016. Cancer Causes Control. 2019;30:1045–55. 10.1007/s10552-019-01217-8. 31428890

[49] Sepassi A, Li M, Zell JA, Chan A, Saunders IM, Mukamel DB. Rural-urban disparities in colorectal cancer screening, diagnosis, treatment, and survivorship care: a systematic review and meta-analysis. Oncologist. 2024;29:e431-46. 10.1093/oncolo/oyad347. PMC10994268 38243853

[50] Doogan NJ, Roberts ME, Wewers ME, Stanton CA, Keith DR, Gaalema DE, et al. A growing geographic disparity: rural and urban cigarette smoking trends in the United States. Prev Med. 2017;104:79–85. 10.1016/j.ypmed.2017.03.011. PMC5600673 28315761

[51] Owusu DN, Mensah EA, Mamudu S, Brooks B, Shoham D. Rural-urban disparities in colorectal cancer screening in United States: Blinder-Oaxaca decomposition analysis of BRFSS data. Cancer Causes Control. 2025;36:1911–7. 10.1007/s10552-025-02071-7. 40971095

[52] Rohatgi KW, Marx CM, Lewis-Thames MW, Liu J, Colditz GA, James AS. Urban-rural disparities in access to low-dose computed tomography lung cancer screening in Missouri and Illinois. Prev Chronic Dis. 2020;17:E140. 10.5888/pcd17.200202. PMC7665516 33155970

[53] Cui P, Huang Y, Han J, Song F, Chen K. Ambient particulate matter and lung cancer incidence and mortality: a meta-analysis of prospective studies. Eur J Public Health. 2015;25:324–9. 10.1093/eurpub/cku145. 25201901

[54] Bhatia S, Landier W, Paskett ED, Peters KB, Merrill JK, Phillips J, et al. Rural–urban disparities in cancer outcomes: opportunities for future research. J Natl Cancer Inst. 2022;114:940–52. 10.1093/jnci/djac030. PMC9275775 35148389

[55] Hirko KA, Xu H, Rogers LQ, Martin MY, Roy S, Kelly KM, et al. Cancer disparities in the context of rurality: risk factors and screening across various U.S. rural classification codes. Cancer Causes Control. 2022;33:1095–105. 10.1007/s10552-022-01599-2. PMC9811397 35773504

[56] Singh GK, Jemal A. Socioeconomic and racial/ethnic disparities in cancer mortality, incidence, and survival in the United States, 1950–2014: over six decades of changing patterns and widening inequalities. J Environ Public Health. 2017. 10.1155/2017/2819372. PMC5376950 28408935

[57] Eom KY, Berg KA, Joseph NE, Runner K, Tarabichi Y, Khiyami A, et al. Neighborhood and racial influences on triple negative breast cancer: evidence from Northeast Ohio. Breast Cancer Res Treat. 2023;198:369–81. 10.1007/s10549-023-06883-6. PMC10716786 36781520

[58] Fowler ME, Reeves HD, Smith MJ, Saini G, Bhargava M, Aneja R. Structural determinants of racial disparities in breast cancer survival in Alabama. Cancer Epidemiol Biomarkers Prev. 2025;34:1740–8. 10.1158/1055-9965.EPI-25-0195. PMC12323589 40632137

[59] Luningham JM, Seth G, Saini G, Bhattarai S, Awan S, Collin LJ, et al. Association of race and area deprivation with breast cancer survival among Black and White women in the State of Georgia. JAMA Netw Open. 2022;5:e2238183. 10.1001/jamanetworkopen.2022.38183. PMC9617173 36306134

[60] Gupta S, Coronado GD, Argenbright K, Brenner AT, Castañeda SF, Dominitz JA, et al. Mailed fecal immunochemical test outreach for colorectal cancer screening: summary of a Centers for Disease Control and Prevention–sponsored Summit. CA Cancer J Clin. 2020;70:283–98. 10.3322/caac.21615. PMC7523556 32583884

[61] Jefford M, Howell D, Li Q, Lisy K, Maher J, Alfano CM, et al. Improved models of care for cancer survivors. Lancet. 2022;399:1551–60. 10.1016/S0140-6736(22)00306-3. PMC9009839 35430022

[62] Battaglia TA, Zhang X, Dwyer AJ, Rush CH, Paskett ED. Change agents in the oncology workforce: let’s be clear about community health workers and patient navigators. Cancer. 2022;128:2664–8. 10.1002/cncr.34194. PMC9201990 35699614

[63] Jiang C, Yabroff KR, Deng L, Wang Q, Perimbeti S, Shapiro CL, et al. Self-reported Transportation Barriers to Health Care Among US Cancer Survivors. JAMA Oncol. 2022;8:775–8. 10.1001/jamaoncol.2022.0143. PMC8949758 35323841

[64] Shaffer KM, Turner KL, Siwik C, Gonzalez BD, Upasani R, Glazer JV, et al. Digital health and telehealth in cancer care: a scoping review of reviews. Lancet Digital Health Elsevier. 2023;5:e316–27. 10.1016/S2589-7500(23)00049-3. PMC10124999 37100545

[65] Salmani H, Ahmadi M, Shahrokhi N. The impact of mobile health on cancer screening: a systematic review. Cancer informatics. SAGE Publications Sage UK: London, England; 2020;19:1176935120954191. 10.1177/1176935120954191 PMC7573752 33116352

[66] Chan RJ, Milch VE, Crawford-Williams F, Agbejule OA, Joseph R, Johal J, et al. Patient navigation across the cancer care continuum: an overview of systematic reviews and emerging literature. CA Cancer J Clin. 2023;73:565–89. 10.3322/caac.21788. 37358040

[67] Liu A, Garcia-Torres LC, Johnson C, Haver MK, Gwede CK, Christy SM. Cancer screening educational interventions in rural and farmworker communities: a systematic literature review. Ethn Health. 2023;28:335–57. 10.1080/13557858.2022.2056145. PMC9626390 35499269

[68] Wercholuk AN, Parikh AA, Snyder RA. The road less traveled: transportation barriers to cancer care delivery in the rural patient population. JCO Oncol Pract. 2022;18:652–62. 10.1200/OP.22.00122. 35834768

[69] Freni-Sterrantino A, Ventrucci M, Rue H. A note on intrinsic conditional autoregressive models for disconnected graphs. Spat Spatiotemporal Epidemiol. 2018;26:25–34. 10.1016/j.sste.2018.04.002. 30390932

[70] Cheng E, Soulos PR, Irwin ML, Cespedes Feliciano EM, Presley CJ, Fuchs CS, et al. Neighborhood and individual socioeconomic disadvantage and survival among patients with nonmetastatic common cancers. JAMA Netw Open. 2021;4:e2139593. 10.1001/jamanetworkopen.2021.39593. PMC8683967 34919133

[71] DeGuzman PB. Identifying Barriers to Navigation Needs of Cancer Survivors in Rural Areas. 2015 [cited 2025 Nov 30]; https://www.jons-online.com/issues/2015/october-2015-vol-6-no-5/1357-identifying-barriers-to-navigation-needs-of-cancer-survivors-in-rural-areas. Accessed 30 Nov 2025

[72] CDC. Cancer Prevention: Rural Policy Brief [Internet]. 2024. https://www.cdc.gov/rural-health/php/policy-briefs/cancer-policy-brief.html

[73] Ho TD, Morris B, Tatum KL, Glasgow TE, Barsell DJ, Fugate-Laus K, et al. Differences in lifestyle behaviors, quality of life, and access to healthcare among rural and urban cancer survivors. Prev Oncol Epidemiol. 2024;2:2374967. 10.1080/28322134.2024.2374967.

[74] Adelson K, Rocque G. Community health worker navigation for patients with cancer: It is time to scale up. J Clin Oncol. 2024;42:491–3. 10.1200/JCO.23.01723 38175984

[75] CDC. Cancer Survivors: Tailor Program Services to Local Needs and Resources [Internet]. 2024. https://www.cdc.gov/cancer-survivors/hcp/rural/tailor-services.html

[76] Argenbright K, Berry E. Innovative cancer survivorship services for rural and underserved communities. J Natl Cancer Inst Monogr. 2021;2021:31–4. 10.1093/jncimonographs/lgab001. 34478508

