# Correction to: Neighborhood geographic disparities in colorectal, prostate, breast, and lung cancer risk in Alabama

**DOI:** 10.1007/s11764-026-02048-8

**Published:** 2026-06-02

**Authors:** R. Blake Buchalter, Nashira I. Brown, Mahak Bhargava, Hayden D. Reeves, Syed M. Qasim Hussaini, Timiya S. Nolan, Ritu Aneja, Mackenzie E. Fowler

**Affiliations:** 1 Department of Epidemiology, School of Public Health, University of Alabama at Birmingham, Birmingham, AL USA; 2 O’Neal Comprehensive Cancer Center, Heersink School of Medicine, University of Alabama at Birmingham, Birmingham, AL USA; 3 Department of Health Behavior, School of Public Health, University of Alabama at Birmingham, Birmingham, AL USA; 4 Department of Nutrition Sciences, School of Health Professions, University of Alabama at Birmingham, Birmingham, AL USA; 5 Division of Hematology & Oncology, Department of Medicine, University of Alabama at Birmingham, Birmingham, AL USA; 6 Division of General Internal Medicine and Population Science, Department of Medicine, University of Alabama at Birmingham, Birmingham, AL USA

**Correction to: Journal of Cancer Survivorship**

**https://doi.org/10.1007/s11764-026-02026-0**

In this article, the author’s name “Mahak Bhargava” was incorrectly written with degree as “Mahak Bhargava BTech.”

The original article has been corrected.

