# Supplementary material for: Neighborhood geographic disparities in colorectal, prostate, breast, and lung cancer risk in Alabama
Source: J Cancer Surviv. 2026 May 2;20(Suppl 2):1936–46. doi: 10.1007/s11764-026-02026-0 (PMC13230096; doi:10.1007/s11764-026-02026-0)
Supplement: Supplementary file 1 [file 11764_2026_2026_MOESM1_ESM.pdf]

Supplementary Material

Table S1: BYM2 model results for colorectal cancer incidence in Alabama census tracts

Parameter	*Median (95% CrI)*
Fixed effects	
Exponentiated intercept (RR)	**1.022 (1.002-1.043)**
Spatial random effects	
φ (structured variance proportion)	0.720 (0.192-0.968)
τ (precision)	9.057 (4.818-14.467)
σ total	0.332 (0.263-0.454)
σ structured	0.282 (0.116-0.447)
σ unstructured	0.176 (0.047-0.408)

DIC: 7485
Bold: Fixed effects significant at 95% CrI

Table S2: BYM2 model results for prostate cancer incidence in Alabama census tracts

Parameter	*Median (95% CrI)*
Fixed effects	
Exponentiated intercept (RR)	**1.030 (1.008-1.053)**
Spatial random effects	
φ (structured variance proportion)	0.952 (0.818-0.996)
τ (precision)	11.40 (7.640-16.324)
σ total	0.297 (0.248-0.361)
σ structured	0.289 (0.224-0.360)
σ unstructured	0.065 (0.016-0.154)

DIC: 8640
Bold: Fixed effects significant at 95% CrI

Table S3: BYM2 model results for breast cancer incidence in Alabama census tracts

Parameter	*Median (95% CrI)*
Fixed effects	
Exponentiated intercept (RR)	1.011 (0.994-1.029)
Spatial random effects	
φ (structured variance proportion)	1.000 (0.999-1.000)
τ (precision)	26.00 (1.540-41.92)
σ total	0.196 (0.155-0.254)
σ structured	0.196 (0.155-0.254)
σ unstructured	0.0008 (0.0004-0.0015)

DIC: 8590
Bold: Fixed effects significant at 95% CrI

Table S4: BYM2 model results for lung cancer incidence in Alabama census tracts i

Parameter	*Median (95% CrI)*
Fixed effects	
Exponentiated intercept (RR)	0.993 (0.975-1.014)
Spatial random effects	
φ (structured variance proportion)	0.760 (0.244-0.967)
τ (precision)	4.094 (2.514-5.729)
σ total	0.494 (0.418-0.629)
σ structured	0.431 (0.206-0.618)
σ unstructured	0.242 (0.076-0.547)

DIC: 8283
Bold: Fixed effects significant at 95% CrI

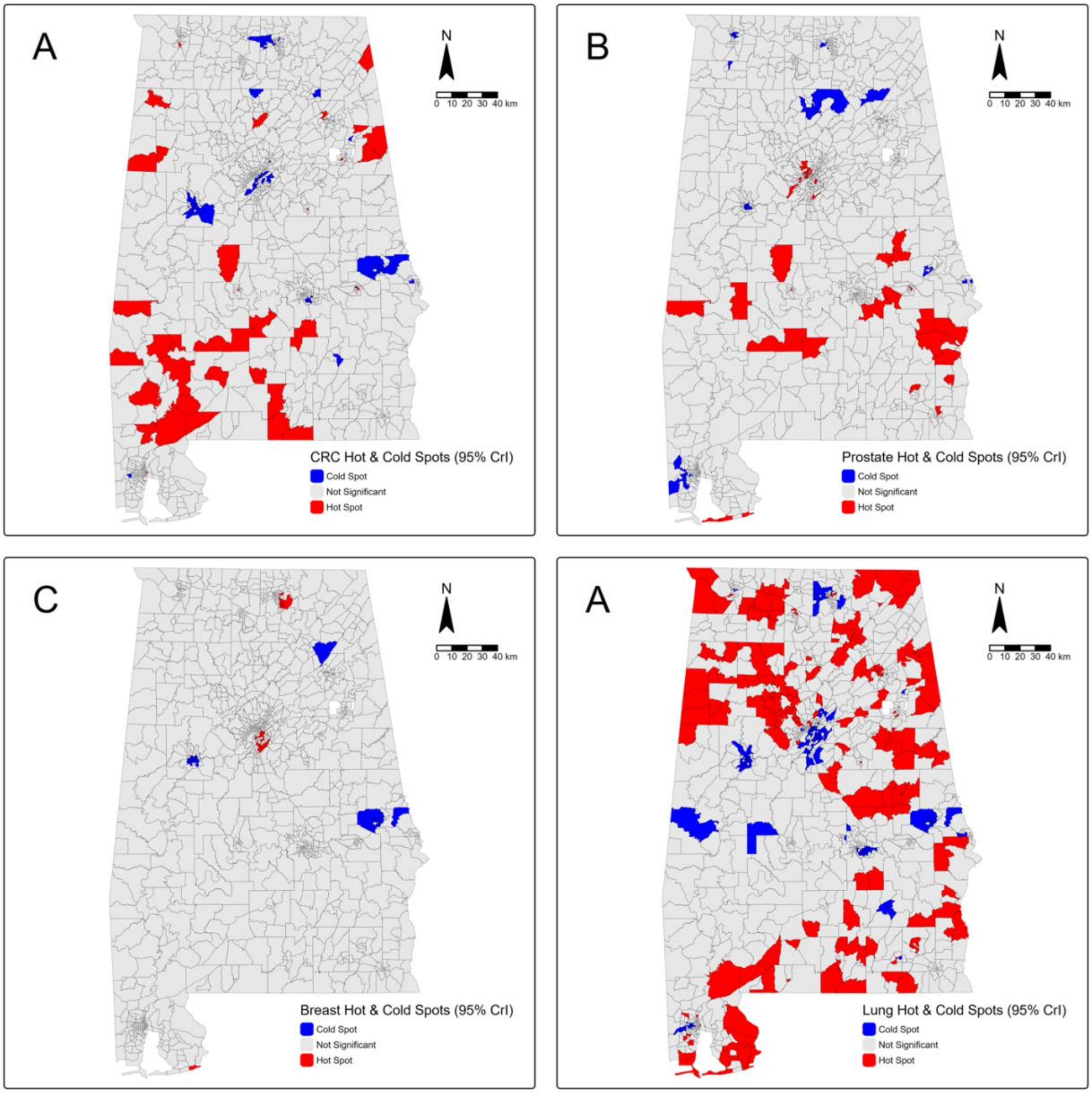

Figure S1. Hot and cold spots maps for A) colorectal cancer, B) prostate cancer, C) breast cancer, and D) lung cancer incidence at 95% CrI in Alabama census tracts.

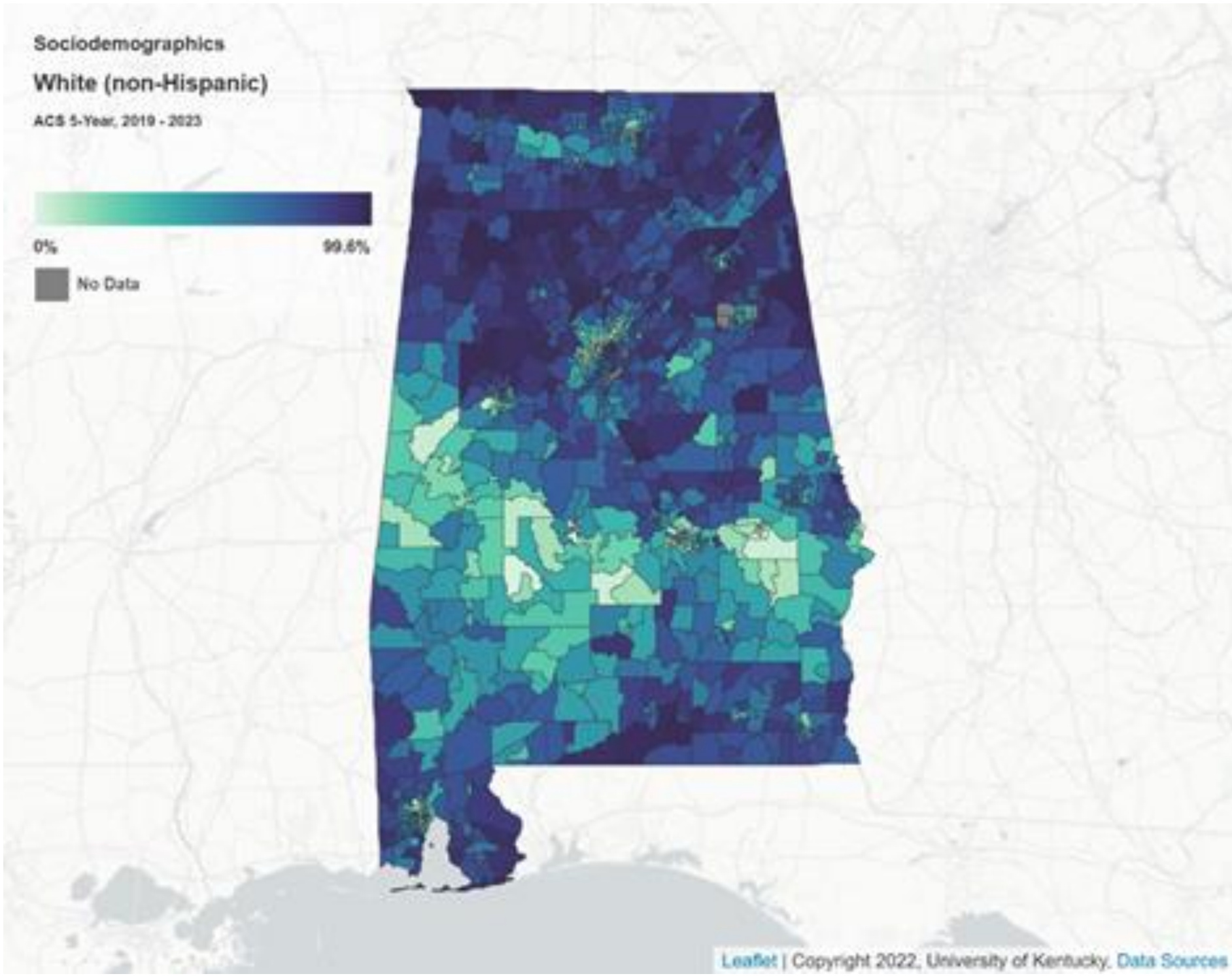

Figure S2. Distribution of non-Hispanic White Population across Alabama Census Tracts.

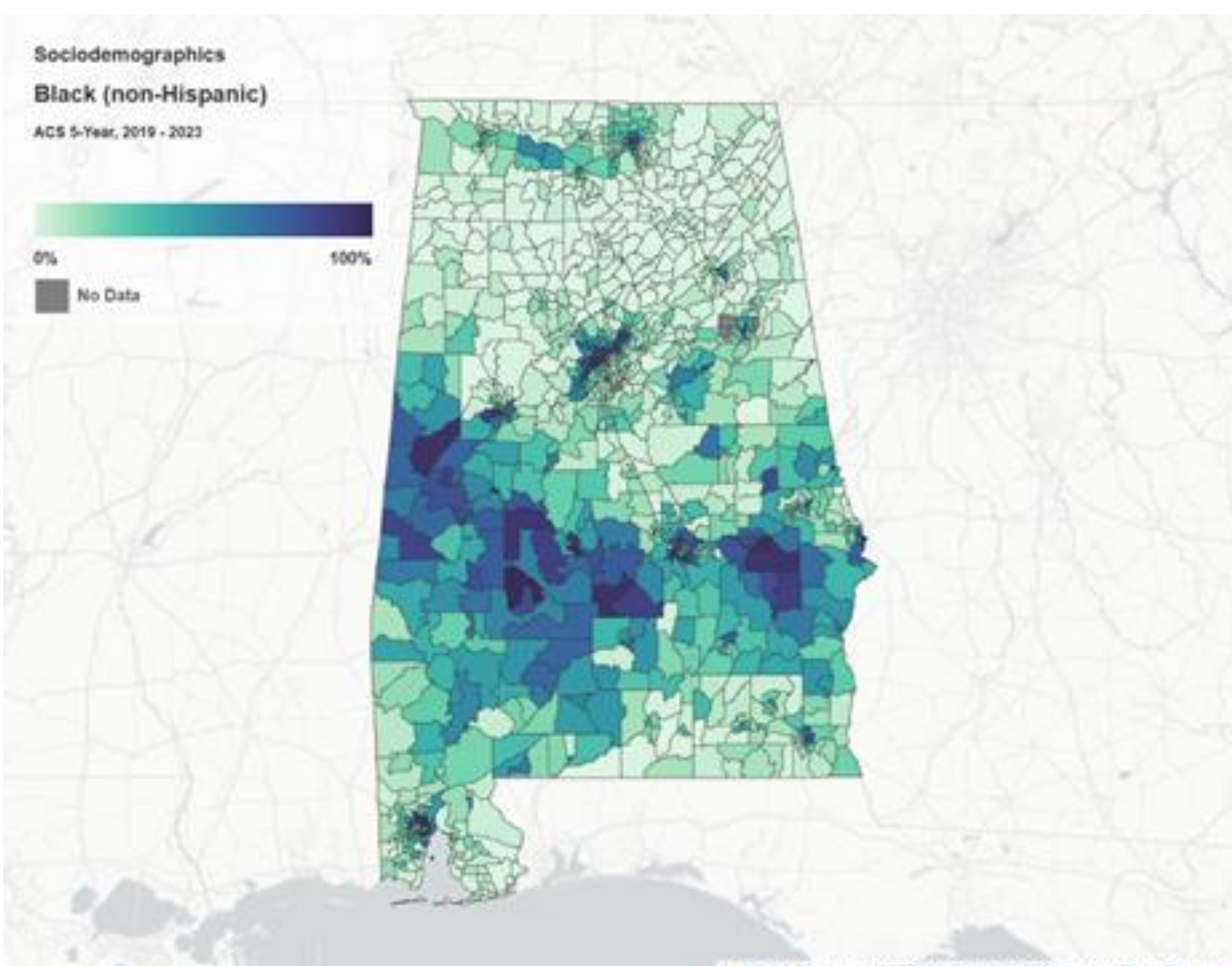

Figure S3. Distribution of non-Hispanic Black Population across Alabama Census Tracts.

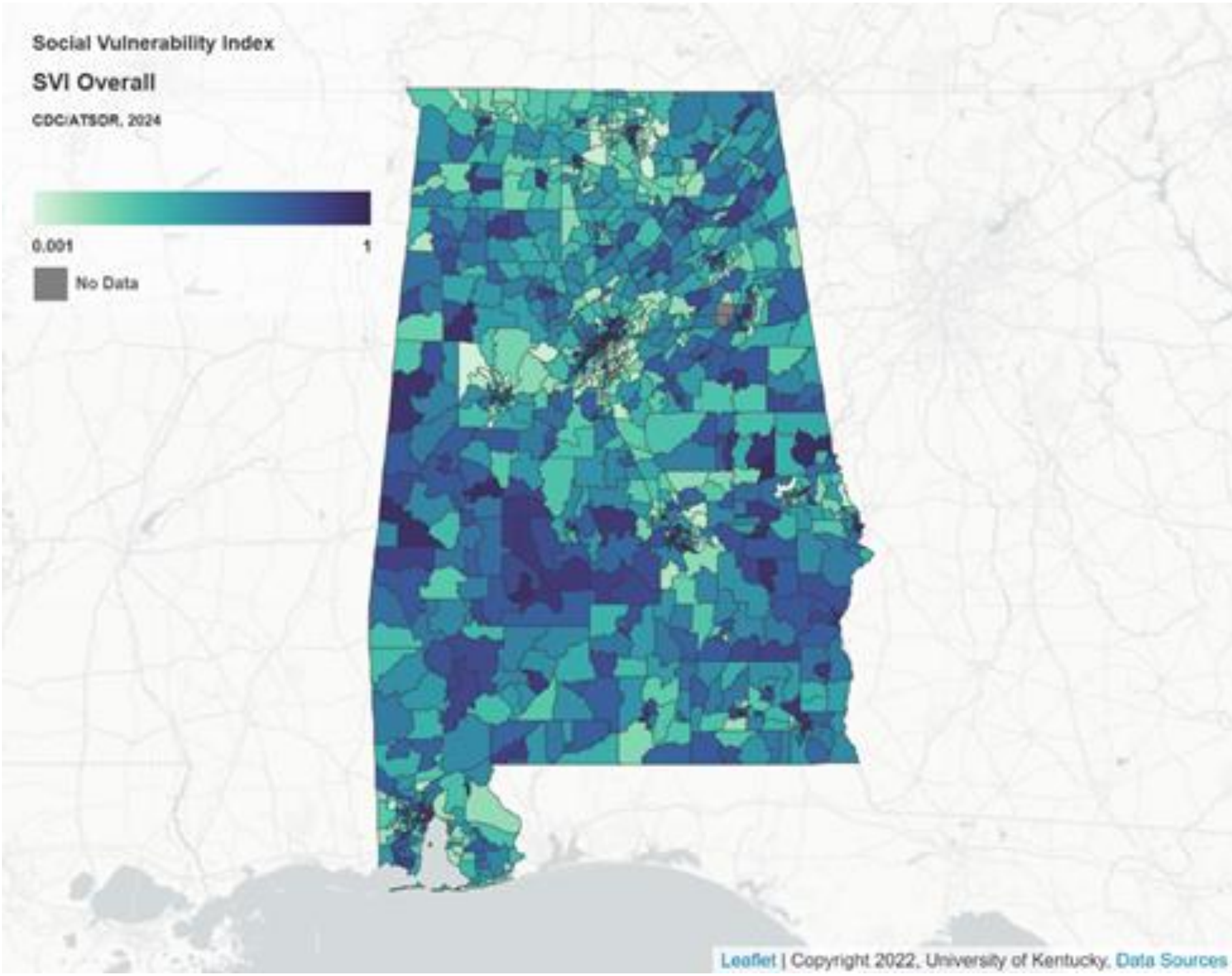

Figure S4. Proportion of Adults who Currently Smoke across Alabama Census Tracts.

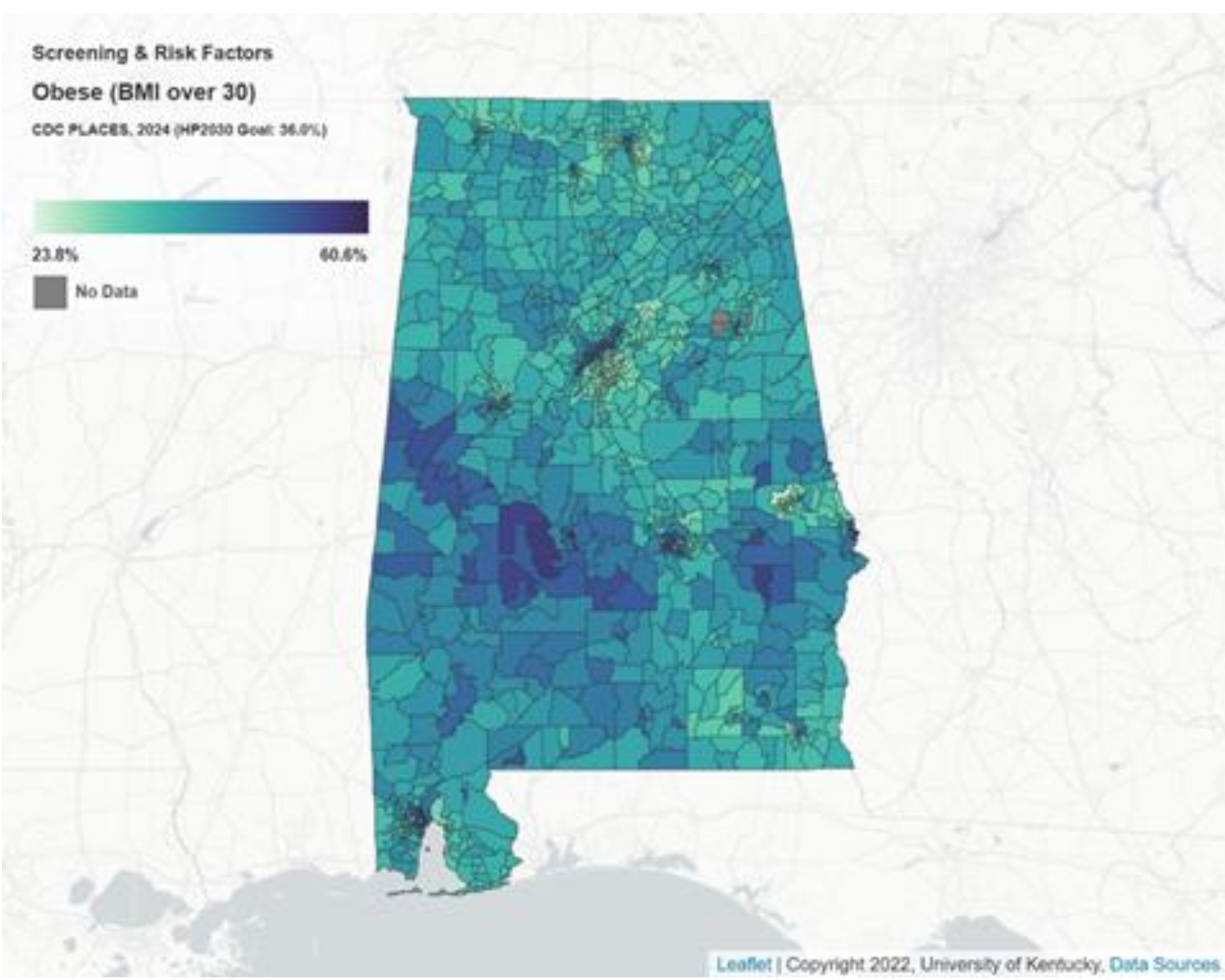

Figure S5. Proportion of Adults with Obese Body Mass Index across Alabama Census Tracts.

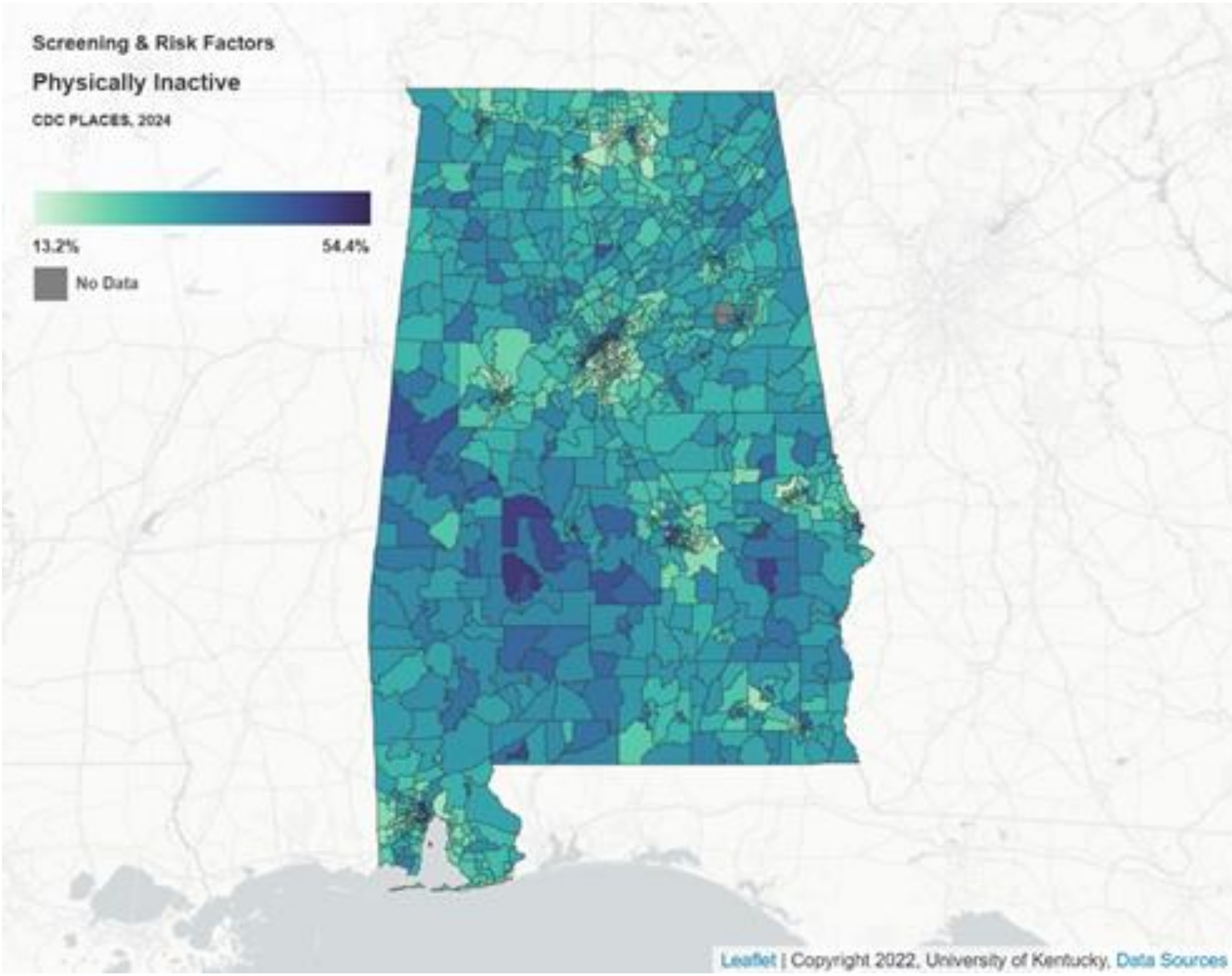

Figure S6. Proportion of Adults who are Physically Inactive across Alabama Census Tracts.

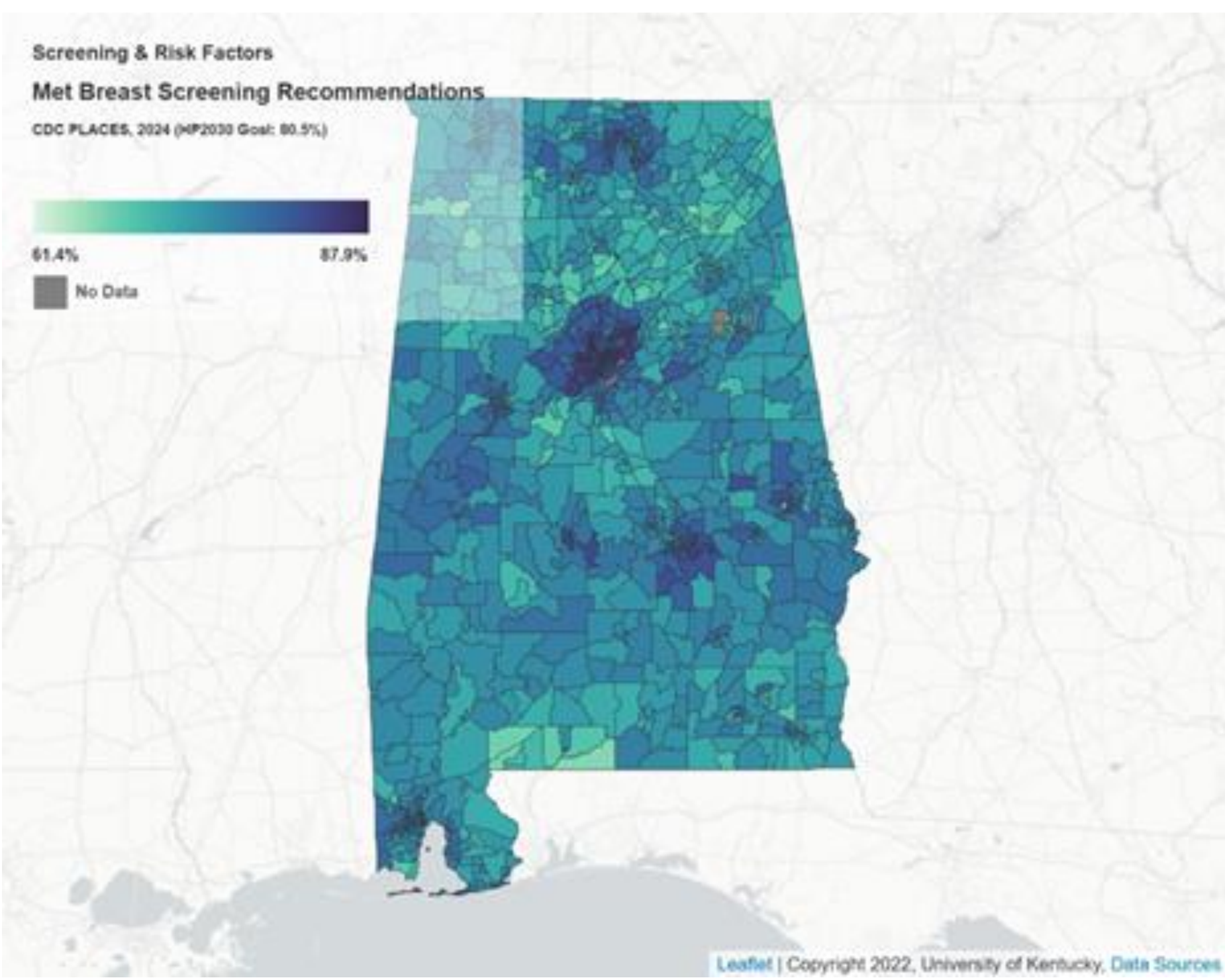

Figure S7. Proportion of Adults Meeting Breast Cancer Screening Recommendations across Alabama Census Tracts.

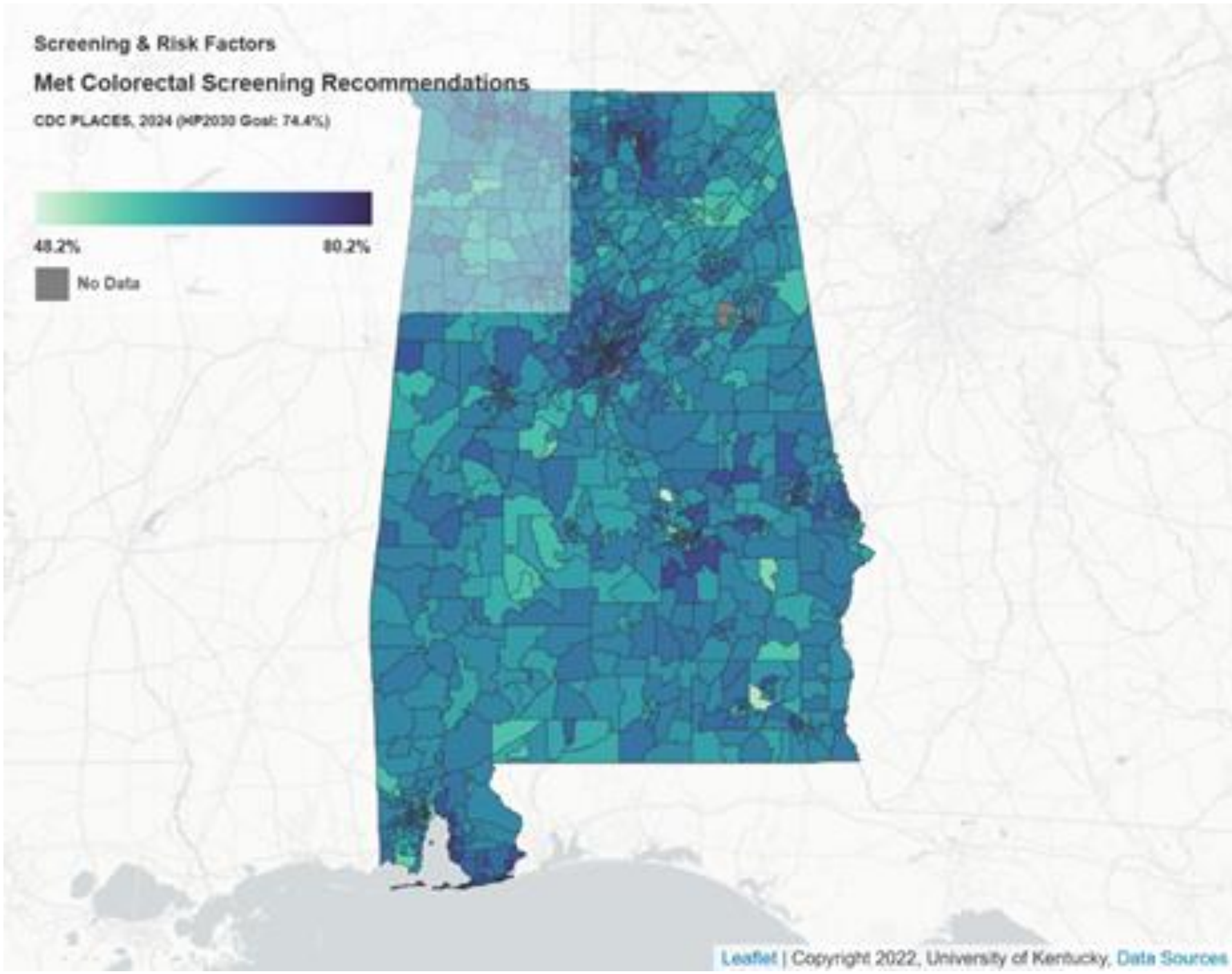

Figure S8. Proportion of Adults Meeting Colorectal Cancer Screening Recommendations across Alabama Census Tracts.

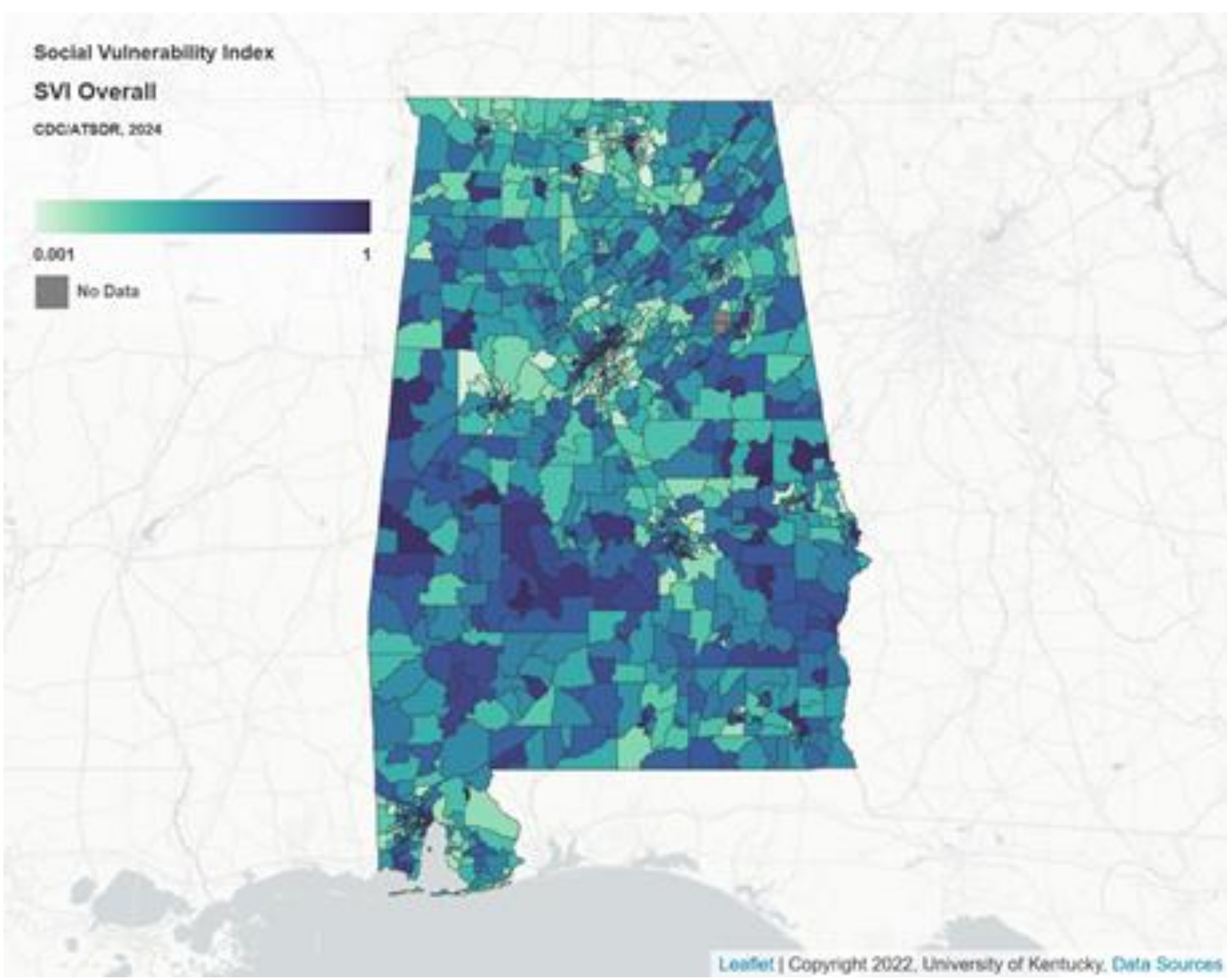

Figure S9. Distribution of Social Vulnerability Index (SVI) across Alabama Census Tracts. SVI is a similar metric to ADI, ranging from 0 to 1 with 1 indicating the highest level of vulnerability in the census tract.

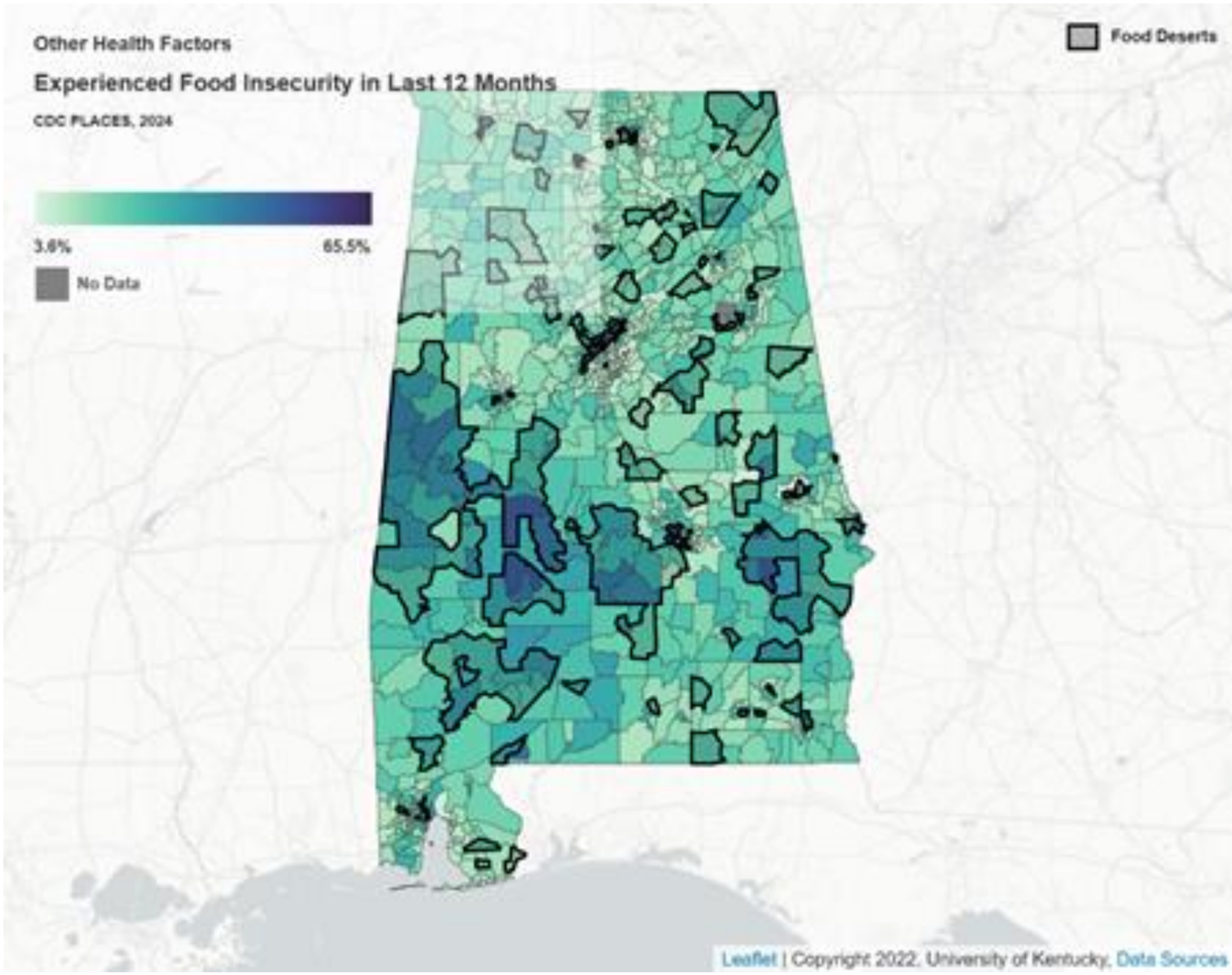

Figure S10. Proportion Experiencing Food Insecurity in the Past 12 Months across Alabama Census Tracts overlaid with Food Deserts.

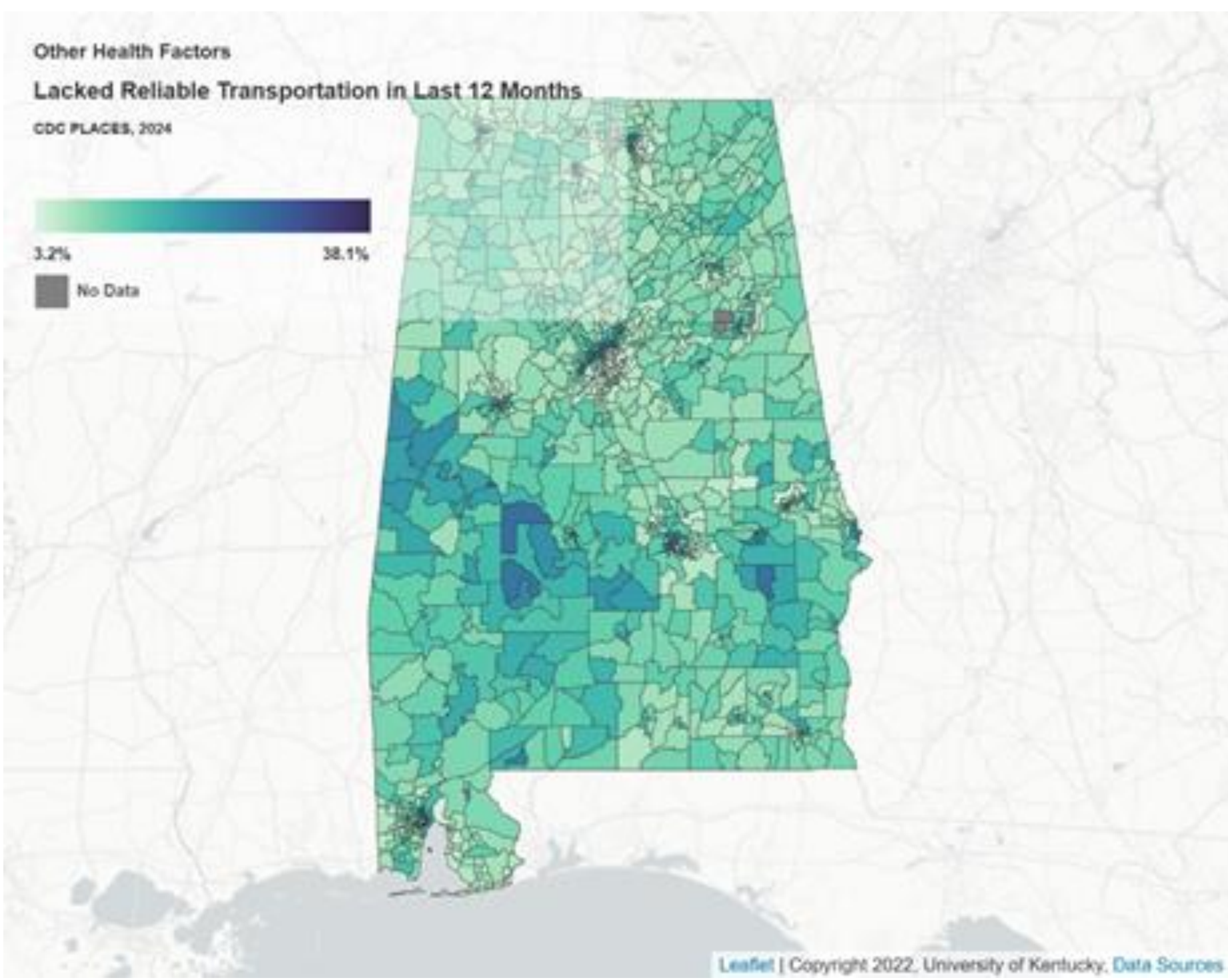

Figure S11. Proportion Lacking Reliable Transportation in the Past 12 Months across Alabama Census Tracts.

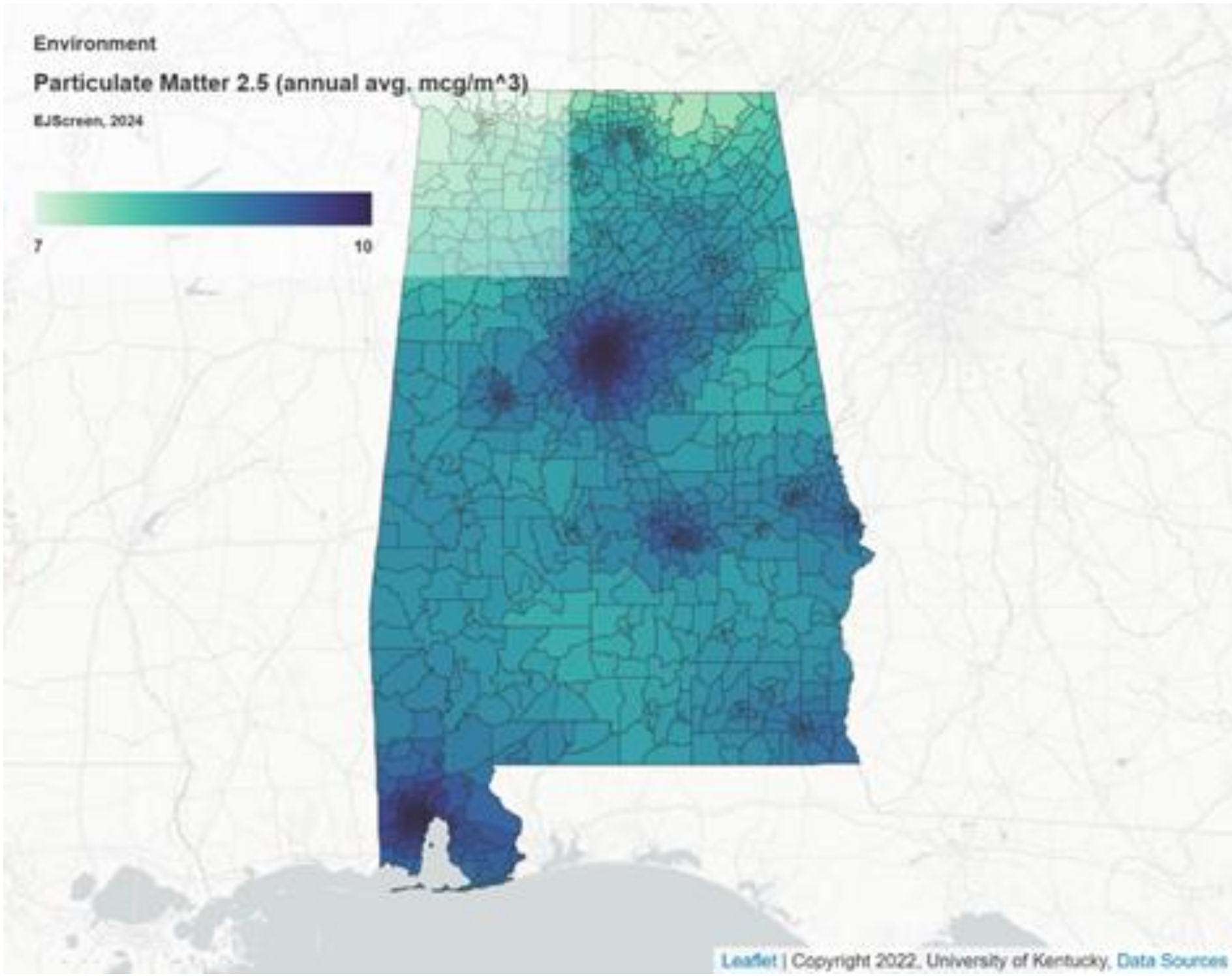

Figure S12. Distribution of Particulate Matter 2.5 (annual average, mcg/m^3) across Alabama Census Tracts.

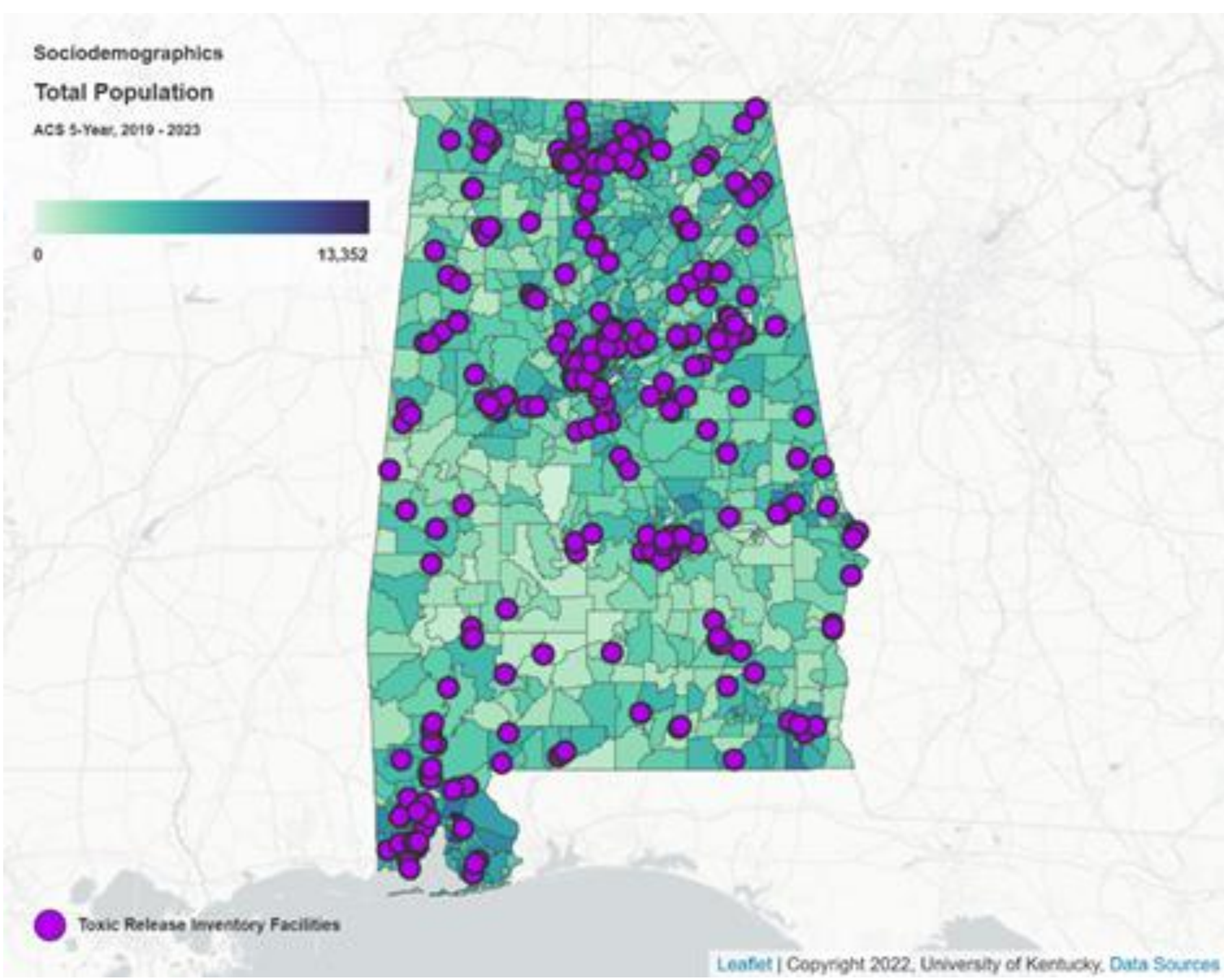

Figure S13. Distribution of Total Population in Alabama overlaid with Locations of Toxic Release Inventory Facilities.
